# Optimal sizing and energy scheduling of isolated microgrid considering the battery lifetime degradation

**DOI:** 10.1371/journal.pone.0211642

**Published:** 2019-02-14

**Authors:** Muhammad Sufyan, Nasrudin Abd Rahim, ChiaKwang Tan, Munir Azam Muhammad, Siti Rohani Sheikh Raihan

**Affiliations:** 1 Higher Institution Centre of Excellence (HICoE), UM Power Energy Dedicated Advanced Centre (UMPEDAC) Wisma R & D, University of Malaya, Kuala Lumpur, Malaysia; 2 Iqra University, Main Campus, Karachi, Pakistan; 3 Department of Electrical Engineering, University of Malaya, Kuala Lumpur, Malaysia; Northeast Electric Power University, CHINA

## Abstract

The incessantly growing demand for electricity in today’s world claims an efficient and reliable system of energy supply. Distributed energy resources such as diesel generators, wind energy and solar energy can be combined within a microgrid to provide energy to the consumers in a sustainable manner. In order to ensure more reliable and economical energy supply, battery storage system is integrated within the microgrid. In this article, operating cost of isolated microgrid is reduced by economic scheduling considering the optimal size of the battery. However, deep discharge shortens the lifetime of battery operation. Therefore, the real time battery operation cost is modeled considering the depth of discharge at each time interval. Moreover, the proposed economic scheduling with battery sizing is optimized using firefly algorithm (FA). The efficacy of FA is compared with other metaheuristic techniques in terms of performance measurement indices, which are cost of electricity and loss of power supply probability. The results show that the proposed technique reduces the cost of microgrid and attain optimal size of the battery.

## 1. Introduction

In the last few decades, the world is seeing an unprecedented rise in its population with the resultant subsequent excessive power demand, both of which are the main operative factors behind global warming and carbon emissions. Unfortunately, we are still adamantly depending on the usage of fossil fuels which incidentally are still playing the major role in supplying energy for the power generation and transportation system. However, continual and inevitable depletion of fossil fuel resources in the recent years has put a serious pressure to bear on the governments and energy entrepreneurs to be responsible enough to move towards replenishment of energy through RES [1]. Greenhouse gas emission is reduced by replacing fossil fuels with renewable energy and leads to a growth in the industrial sector. However, the intermittent nature of RES is thwarting the stability of the power system in the economic sense of the word, hence, efficiently controlled methods have become the order of the day to overcome the issues of voltage disturbances, frequency regulations and network security during the high penetration of the RES to meet the growing demand of the population at large [2]. Table 1 shows all the nomenclature that would be used in this paper.

**Table 1:** Nomenclature table.

Indices		Variables	
*t*	time period (h)	*P*_*w*,*h*_	output power of wind turbine at hour h (kW)
*i*	diesel generator	*IC*_*WT*_	initial cost of wind turbine ($/kW)
		*C*_*WT*_	cost of wind turbine power ($)
Parameters		*P*_*PV*,*out*_	output power of the solar panel (kW)
*p*_*w*,max_	maximum power output of WT (kW)	*IC*_*PV*_	initial cost of photovoltaic ($/kW)
*v*_*h*_	speed of wind at hour h (m/s)	*C*_*PV*_	cost of solar photovoltaic power ($)
*v*_*rt*_	rated wind speed (m/s)	*C*_*d*.*gen*_	cost of diesel generator ($)
*v*_*c*,*i*_	cut-in wind speed (m/s)	*C*_*BATT*_	cost of energy storage ($)
*v*_*c*,*o*_	cut-out wind speed (m/s)	*P*_*L*_	microgrid load (kW)
*i*_*r*_	interest rate	*SC*_*D*_	daily scheduling cost ($)
*Ly*	projected lifetime	MC	maintenance cost ($/kWh)
*P*_*PV*,*rated*_	maximum power of PV (kW)	TCPD	total cost per day ($)
*I*	solar radiation at particular day(W/m^2^)	Acronyms	
*I*_*ref*_	solar radiation at standard temperature	RES	Renewable Energy Sources
*T*_*ref*_	standard temperature	DER	distributed energy resources
*T*_*amb*_	ambient temperature of the solar panel	ESS	energy storage system
*k*_*t*_	coefficient of solar power	PV	photovoltaic
*P*_*d*.*gen*,*i*_	power generated by *i*^th^ generator at time *t* (kW)	WT	wind turbine
*a*_*i*_,*b*_*i*_,*c*_*i*_	cost coefficients of the *i*^th^ generators	BESS	battery energy storage system
*C*_*batt*,*cap*_	capital cost of energy storage ($)	LOLE	loss of load expectation
*P*_*batt*_	amount of power charged/discharged by battery	TOU	time of use
*E*_*batt*,*t*_	battery energy storage total capacity	VRB	vanadium redox battery
*l*_*c*_(*DOD*_*batt*_)	number of cycles of energy storage at particular DOD	MILP	mixed integer linear programming
*η*_*batt*_	efficiency of the energy storage	DOD	depth of discharge
*P*_*batt*,*ch*_	amount of power charged by energy storage	CRF	capital recovery factor
*P*_*batt*,*dch*_	amount of power discharged by energy storage	SOC	state of charge
$\eta_{batt}^{ch}$/$\eta_{batt}^{dch}$	battery charging /discharging efficiency	COE	cost of electricity
$E_{batt}^{\min}$/$E_{batt}^{\max}$	minimum /maximum battery capacity level	LPSP	loss of power supply probability
*μ*_*batt*,*st*_	binary status of battery operation	OC	operating cost
$P_{d.gen,i}^{\min}$/$P_{d.gen,i}^{\max}$	minimum and maximum generator limit		

Microgrids have emerged as a platform to integrate DER, such as diesel generators (DG), wind turbine (WT), microturbine (MT), fuel cell (FC), solar photovoltaic (PV) panels and energy storage system (ESS) within a network to feed into the utility grid in a more orderly and manageable network. Microgrids can be operated in the islanded mode as well as grid-connected mode, depending on the load conditions and electricity market price [3, 4], providing the potential to solve the existing power system problems of stability, reliability and demand response.

Because of the limited reach of utility grid, microgrids in islanded mode are more intended for the power balance as compared to the grid-connected mode. Therefore, reliable power sources like synchronous generator and energy storage are the crucial elements to regulate voltage and frequency and improve the stability of the system [5, 6]. Over the past few years, ESS has become an essential component of microgrid. The voltage and frequency regulation in an islanded microgrid can be performed by ESS in the absence of synchronous generators. Moreover, the power fluctuations caused by RES can also be reduced by ESS. In addition, ESS is capable of storing the energy during the periods of high-power generation and releasing it when the load exceeds the power generation capacity. ESS with high energy density and longer discharge time are utilized for the applications related to economic energy dispatch and peak shaving. On the other hand, a high power density ESS with a fast response capability is employed for the voltage control and frequency regulation applications [7, 8].

Battery energy storage systems are best suited for power system applications due to their technical benefits and ability to provide both the power and the energy density. In order to ensure the reliability, security and economic benefits of the microgrid, ascertaining an optimal size of a BESS is very much essential. Economic scheduling together with an optimal battery size is also significant for rural electrification schemes in small towns where the electrical grid is not available. The operation and the scheduling of the BESS have been addressed by many researchers but the design and estimate of its optimal size to achieve a cost-effective system with minimum power losses is still in progress.

In [9], the energy storage size is determined for the frequency regulation services in an islanded microgrid. The overloading characteristic of BESS is implemented for a short time duration to control frequency, resulting in a quick response of battery to overcome a power mismatch. However, the authors did not consider the impact of lifetime degradation and economic drawbacks by overloading the BESS.

A load shedding scheme for ascertaining an optimal battery size is considered in [10]. The proposed method has made use of a metaheuristic optimization algorithm to control the frequency and minimize the operating cost of the microgrid. A unit commitment approach for the sizing of energy storage system in grid-connected and islanded mode is analyzed in [11]. A typical load profile and renewable energy data have been considered for the BESS sizing to reduce the total cost and increase the economic benefits of the microgrid. The effect of uncertainty of renewable energy on BESS and scheduling of DER are optimized by here and now approach. A multi-objective optimization problem for the optimal location and sizing of BESS is considered in [12] for the purpose of voltage regulation of the microgrid. The authors observed that total losses of the distribution system along with the BESS installation cost and investment cost of the DER are minimized. The voltage profiles of the distribution system were improved and the battery lifetime was extended, thereby saving the replacement cost.

A reliability index known as loss of load expectation has been discussed in [13] that helps to curtail the microgrid operating cost by optimizing the battery size. The economic benefits of the microgrid are justified by providing power from ESS to local loads at a low price during peak periods and controlling excessive power generations. A new approach for an optimal energy management using an Alternating Direction Method of the Multiplier (ADMM) has been proposed in [14]. The authors proposed a centralized controller to avoid congestion in the communication network for the data exchange between the customer and the control center. The ADMM algorithm manages the DER of the microgrid for the optimal energy scheduling. The optimal location and size of energy storage was calculated in [15] to reduce the operation cost and LOLE of microgrid. The bi-objective optimization incorporates the demand response program for peak shaving and economic scheduling of the microgrid. A trade-off between the total cost and LOLE yields the optimal size of BESS. Nguyen in [16] claimed that the vanadium redox battery in microgrid system could be effective in both the islanded and the grid-connected modes. The dynamic programming-based unit commitment method is implemented to find the optimum size of the VRB. Furthermore, the authors consider nonlinear charge/discharge efficiencies as a function of voltage, stack efficiency and temperature. A probabilistic approach for optimal battery sizing was analyzed by using the Monte Carlo simulation in [17]. The time of use tariff and the bidirectional energy transfer with the grid has been taken into consideration for an effective battery sizing. However, the profit of installing BESS decreases with the increase in tariff.

A hybrid approach comprising a heuristic and analytical optimization is proposed in [18] to schedule the charge/discharge cycle of BESS in isolated microgrid. The dynamic response is implemented under a real time pricing scheme to maintain a balance between the supply and demand in the microgrid and the BESS. The results report the effective participation of BESS in economic scheduling of isolated microgrid. In [19] the uncertainties of load and renewable power generation are mitigated by scheduling the microgrid such that the BESS provides the spinning reserves service. The proposed discretized step transformation method handles the spinning reserve requirement to obtain a balanced tradeoff between the operation cost and the reliability of the microgrid.

The energy scheduling and the cost minimization method in a real time electricity pricing environment are presented in [20]. The energy flow is coordinated in a model predictive control framework considering the TOU with different renewable energy data for the summer and winter periods. The authors suggested the proposed scheme as a benchmark for the real time electricity pricing to minimize the cost consumption with low and high demand periods. The pricing mechanism under a fixed rate, time of use and a real time pricing are compared in a microgrid environment [21, 22]. The results support the real time pricing scheme for the energy selling by distributed generators and energy storage. The information gap decision theory and an interval optimization approach are analyzed to evaluate the risk criteria. In [23], a grey wolf optimization technique has been implemented to determine the optimal BESS size and minimize the microgrid operating cost. The BESS with an initial charge equivalent to its optimal size increases the net profit as compared to the BESS with no charge. A heuristic method incorporating particle swarm optimization (PSO) is used to find the optimal size of the BESS in [24]. The mix-mode energy management strategy is applied to operate the microgrid at the minimum operating cost by integrating three different strategies. Linear programming and MILP methods are used to minimize the cost of the microgrid under these strategies. The genetic algorithm (GA) based method to determine the optimal battery size has been presented in [25]. The proposed method uses the fuzzy expert system to regulate the power flow of the energy storage. A lifetime aging model predicts the lifetime and operating cost of the microgrid.

The lifetime (Ly) of the battery is an essential factor in calculating the annualized cost of BESS, which controls the microgrid operating cost. The lifetime of the battery is affected by two main factors, namely,1) the lifecycle stating the number of charge and discharge cycles a BESS can sustain, and 2) the depth of discharge representing the amount of capacity used by a BESS. In the above literatures, the estimated value of Ly is predicted to calculate the annualized cost of BESS, whereas [25] has used a lifetime prediction model to determine the battery lifetime. However, the calculated lifetime of an optimal battery size is considered to be very short due to deep discharge cycles.

In this paper, a new approach has been presented to schedule the energy resources in the microgrid considering optimal battery size. The addition of energy storage in the microgrid increases capital cost, but also reduces the operating cost of system. Moreover, optimal size of the battery prolongs the lifetime of the storage system and it is significantly affected by the DOD. In this research, to maximize the economic benefit and minimizes the operational cost, microgrid is incorporated with diesel generator, renewable energy and optimal size of energy storage. Therefore, firefly algorithm has been considered to attain the optimal dispatch which minimizes the generation cost. The main goal of the paper is to formulate the battery cost equation for real time analysis considering depth of discharge at each time interval. In addition to that, this research proposes the battery operational cost in term of DOD. Hence optimal DOD, prolongs the life span of BESS. The efficacy of the proposed method has been verified by comparing it with other techniques proposed in the literature. The proposed method manages to attain lower operating cost without the loss of power supply at any interval of time.

The rest of the paper is organized as follows. Section 2 describes the modelling of microgrid and its components. Section 3 presents the power management strategies for the economic scheduling of microgrid and section 4 focuses on the performance measurement indices to evaluate the results. The objective function and the constraints of the system are formulated in section 5. Section 6 describes the methodology of the proposed model to implement the strategies discussed in section 3 with minimal operating cost of microgrid. Section 7 discusses the optimization algorithm whereas simulation results are illustrated in section 8.

## 2. Hybrid microgrid system

**Fig 1:**
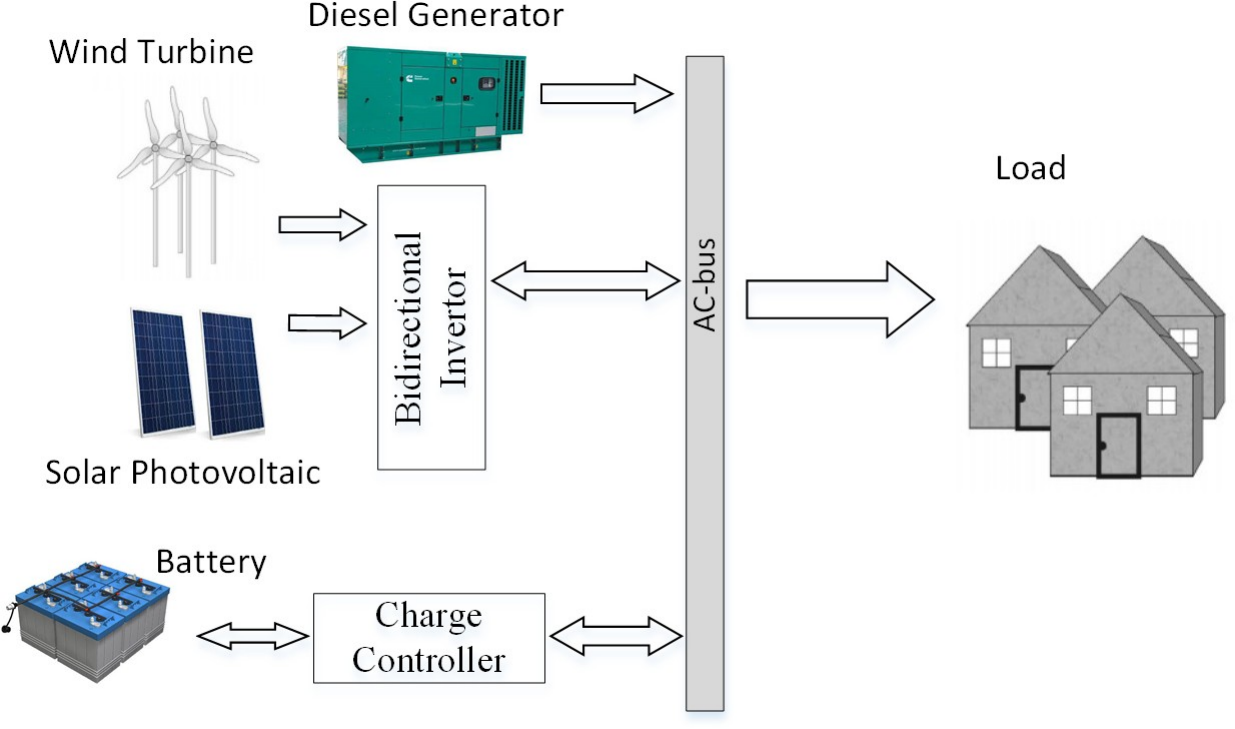
General schematic of hybrid microgrid.

A hybrid isolated microgrid system contains three subsystems: the power demand, the power generation, and the power distribution subsystem. These subsystems have major impact on the cost of the microgrid system. They are dependent on the climatic conditions and the consumer services. This section presents the power and cost models for the wind, solar, diesel generator and energy storage as the DERs of the power generation subsystem, load profile of the residential area as the demand subsystem and the microgrid itself is configured as the power distribution subsystem. The combination of different RESs improves the system efficiency and reduces the requirements of energy storage as compared to a single RES. The general schematic of the microgrid system containing the three systems is as shown in Fig 1.

### 2.1. Wind turbine model

The power model of a wind turbine (WT) measures the power as a function of the hourly wind speed. The relation between the output power and the speed is given by the relationship below [12]:

$$P_{w,h}=\{\begin{array}{cc} 0 & v_{h}\leq v_{c,i}\;or\;v_{h}\geq v_{c,o}\\ p_{w,\max}*\frac{v_{h}-v_{c,i}}{v_{rt}-v_{c,i}} & v_{c,i}\leq v_{h}\leq v_{rt}\\ p_{w,\max} & v_{rt}\leq v_{h}\leq v_{c,o} \end{array}$$ (1)

The cost of power dissipated by wind turbine in a particular day is the product of power dispatched and the initial cost of wind turbine. The capital recovery factor calculates the present value for the 24-hour analysis taking interest rate and projected lifetime into consideration.

$$C_{WT}=\left(\sum_{t=1}^{T}P_{w,h}(t)\right)*IC_{WT}*CRF$$ (2)

$$CRF=\frac{1}{365}\times \frac{i_{r}{\left(1+i_{r}\right)}^{ly}}{{\left(1+i_{r}\right)}^{ly}-1}$$ (3)

### 2.2. Solar PV model

The power measured by solar photovoltaic array is dependent on the solar irradiation and the ambient temperature at each hour. The PV power is given by [26]

$$P_{PV,out}=P_{PV,rated}*\frac{I}{I_{ref}}*\left[1+k_{t}\left\{\left(T_{amb}+(0.0256*I)\right)-T_{ref}\right\}\right]$$ (4)

The cost of power dispatched by solar photovoltaic is dependent on the initial cost and power output defined as

$$C_{PV}=\left(\sum_{t=1}^{T}P_{PV,out}(t)\right)*IC_{PV}*CRF$$ (5)

### 2.3. Diesel generator

A diesel generator and energy storage are the secondary power generation sources for the microgrid when the renewable energy cannot fulfill the required electricity demand. The conventional generator serves as a backup energy source and improves the system reliability by smoothing the power generation from the renewable energy source. The high cost of energy storage has captivated the attention of utility providers to utilize diesel generators in the microgrid. There are a total of three generators considered in this study. The cost of the generator in terms of power dispatch is expressed by [27]

$$C_{d.gen}=F_{i}\left(P_{d.gen}{}_{,i}(t)\right)=a_{i}P_{d.gen,i}^{2}(t)+b_{i}P_{d.gen,i}(t)+c_{i}$$ (6)

### 2.4. Battery energy storage model

**Fig 2:**
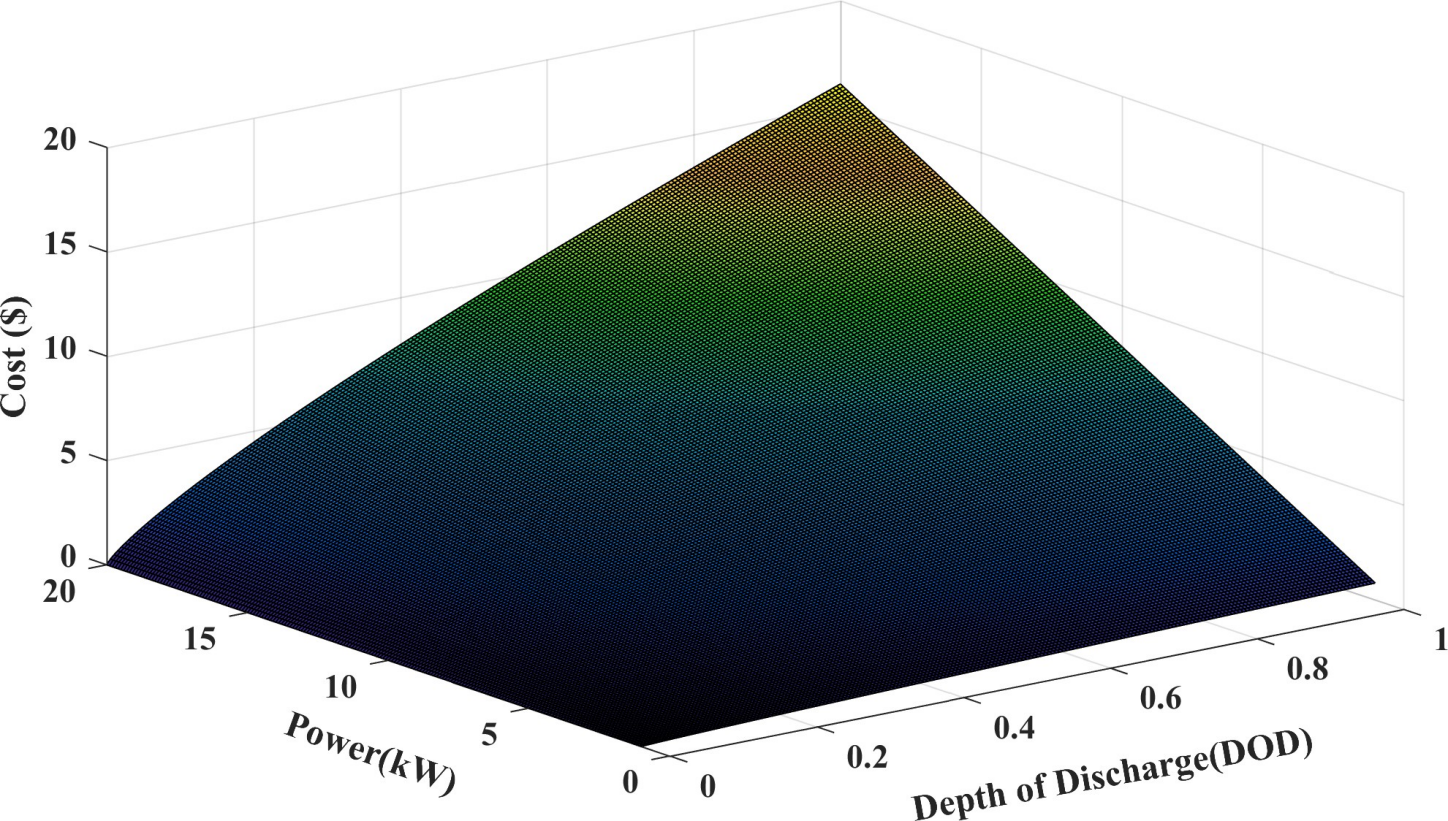
Cost of energy storage for different DOD and power discharge.

The BESS in a microgrid is used to avoid any power mismatch between the demand and generation. The selection of different battery energy storage units, each having its own distinguished characteristics in power and energy, depends on the nature of the power required and the power delivered. Lithium-ion battery is used in this paper as they are currently mainly used for storing wind and solar energy due to its high energy density among other battery technologies, long life cycle and high efficiency [11, 28]. The energy storage cost analysis is shown in Fig 2 for different values of power discharge and depth of discharge. The figure depicts that the cost of the energy storage increases when the DOD is high and similarly the cost is increased when the battery discharges more power. The increase in the discharge power results in decreasing the capacity of the energy storage and causes the DOD to be high. However, the continuous discharge of high power from the energy storage at maximum DOD increases the cost of the energy storage to the maximum value. The cost of charging/discharging battery at any time interval as a function of battery power and DOD is formulated in Eq (7) [29, 30]. The lifecycle of the lithium-ion battery is represented by an exponential function taken from [31]. The cost function of the battery storage during charge/discharge event is modelled as

$$C_{BATT}(t)=\frac{C_{batt,cap}*P_{batt}(t)*\Delta t}{E_{batt,t}*l_{c}(DOD_{batt}(t))*\eta_{batt}{}^{2}}$$ (7)

$$l_{c}(DOD_{batt}(t))=694*{\left(DOD_{batt}(t)\right)}^{-0.795}$$ (8)

$$DOD_{batt}(t)=1-SOC_{batt}(t)$$ (9)

The state of charge (SOC) represents the status of battery capacity given by

$$SOC_{batt}(t+1)=SOC_{batt}(t)-\frac{P_{batt,ch}(t)*\Delta t*\eta_{batt}^{ch}}{E_{batt,t}}-\frac{P_{batt,dch}(t)*\Delta t}{E_{batt,t}*\eta_{batt}^{dch}}$$ (10)

where Δ*t* is the time interval taken as 1 hour. The battery charging $\eta_{batt}^{ch}$ and discharging $\eta_{batt}^{dch}$ efficiency is considered same in this paper and is equal to efficiency of battery.

## 3. Power management strategy

The power management strategy of the microgrid has a direct impact on the operational behavior of the system regardless of grid-connected or isolated mode of operation. However, in the isolated mode the power generated from the distributed resources must satisfy the load demand for a secure and reliable operation; otherwise, the system will face load shedding which will increase the cost in term of power losses. The unavailability of DERs at certain times of the day will force the diesel generator and battery storage to operate and dispatch optimal power. Moreover, an excess power generation by renewable resources necessitates the charging of the battery. The extra energy after charging is dissipated into dump load to avoid overcharging of batteries. Thus, an efficient power management strategy is required to dispatch the power at the lowest cost to reliably serve the load considering the technical constraints of the microgrid. The power strategy for economic scheduling in this paper has been summarized into the following scenarios:

Scenario 1: Renewable energy sources are capable to provide sufficient energy to meet the load demand and the battery is charged by the excess energy.

Scenario 2: This scenario is identical to scenario 1 with the exception that the battery is fully charged and the extra energy generated by renewable sources is dissipated as a dump load.

Scenario 3: Renewable energy resources cannot satisfy the required load of the system. The algorithm will decide to run the diesel generators or discharge the battery depending on the required load and the cost accumulated in two distributed sources.

Scenario 4: The renewable sources energy generation is insufficient to satisfy the required load and depth of discharge of battery storage is high, with the result that the generator will dispatch the remaining power and also to charge the battery to reduce the depth of discharge.

## 4. Performance measurement models

### 4.1 Cost of electricity

The cost of electricity is calculated as an indicator of the economic profitability of hybrid microgrid. The electricity cost is the ratio of the sum of the costs associated with solar photovoltaic (*C*_*PV*_), wind turbine (*C*_*WT*_), diesel generators (*C*_*d*.*gen*_) and the energy storage (*C*_*BATT*_) to the total load of the day. The electricity cost is measured between all the power generation resources and load for the 24-hour analysis.

$$\mathrm{Cost}\;\mathrm{of}\;\mathrm{Electricity}=\frac{C_{PV}+C_{WT}+\sum_{t=1}^{T}\left(C_{d.gen}+C_{BATT}\right)}{\sum_{t=1}^{T}P_{L}}$$ (11)

### 4.2 Reliability analysis

The reliability of the microgrid is measured by the statistical parameter loss of power supply probability. The reliability parameter signifies the probability over the time horizon when the generation fails to satisfy the demand. This failure can be either due to the improper designing of the distributed energy resources, immediate drop in renewable energy or increase in power demand. LPSP can be calculated by either using time series data or by determining the energy accumulative effect over the total load. The latter technique has been used in this paper, as shown by the expression

$$LPSP=\frac{\sum_{t=1}^{T}\left(P_{L}-P_{PV,out}-P_{w,h}-P_{batt,dch}-P_{d.gen}\right)}{\sum_{t=1}^{T}P_{L}}$$ (12)

## 5. Problem formulation

The above-mentioned power management strategy is implemented to obtain an optimal battery size and daily economic scheduling of microgrid. The capital cost of battery constitutes a major factor in calculating the battery size. The optimal BESS sizing is obtained by minimizing the daily scheduling cost of the microgrid and BESS total cost per day. Hence, the objective function of the microgrid is the total operating cost given by the expression

$$OC=SC_{D}+TCPD_{BESS}$$ (13)

$$SC_{D}=Min\sum_{t=1}^{T}\left(\sum_{i=1}^{N}F_{i}\left(P_{d.gen}{}_{,i}(t)\right)\right)+C_{BATT}$$ (14)

$$TCPD_{BESS}=\left(CRF*C_{batt,cap}+\frac{MC}{365}\right)*E_{batt,t}$$ (15)

The scheduling cost for the day is the sum of the cost of three diesel generators dispatching power to fulfil the load demand and the cost of charging/discharging the battery storage. In this study, *N* is taken as three while the time period *T* is formulated as 24 hours. The TCPD of battery storage is the function of battery capital cost and yearly maintenance cost accounted for the lifetime of battery. The optimal battery size will minimize the total cost of microgrid.

### 5.1 Constraints

The energy management operation of the microgrid has been optimized by meeting the following constraints:

#### 5.1.1 ESS constraints

The battery charging and discharging energy is expressed in Eq (16) [11]. The battery discharges when *P*_*batt*_ is positive whereas negative *P*_*batt*_ indicates charging status of BESS. The associated constraint (17) limits the battery power to minimum and maximum value. The charging and discharging of battery depends on the status of the battery power itself, wherein the battery power will be positive in discharging mode and negative in the charging mode. The binary variable *μ*_*batt*,*st*_ states the operating status of the battery. The battery discharges only when *μ*_*batt*,*st*_ is 1 and charges when *μ*_*batt*,*st*_ is 0 avoiding the simultaneous charge and discharge event at any interval. The maximum amount of power charged and discharged by the battery storage during the time *t* is shown by $P_{batt,ch}^{\max}$ and $P_{batt,dch}^{\max}$ respectively. The battery capacity at each interval is within the minimum $E_{batt}^{\min}$ and maximum $E_{batt}^{\max}$ level. The maximum and minimum energy storage capacity in this paper is set as 90% and 15% respectively.

$$E_{batt}(t+1)=\{\begin{array}{cc} E_{batt}(t)-\frac{P_{batt,dch}(t)*\Delta t}{\eta_{batt}^{dch}} & \left(P_{batt}(t)>0\right)\\ E_{batt}(t)-P_{batt,ch}(t)*\Delta t*\eta_{batt}^{ch} & \left(P_{batt}(t)<0\right) \end{array}$$ (16)

$$P_{batt}^{min}\leq P_{batt}(t)\leq P_{batt}^{max}$$ (17)

$$0\leq P_{batt,dch}(t)\leq P_{batt}*\mu_{batt,st}$$ (18)

$$-P_{batt}*(1-\mu_{batt,st})\leq P_{batt,ch}(t)\leq 0$$ (19)

$$E_{batt}^{\min}\leq E_{batt}(t)\leq E_{batt}^{\max}$$ (20)

#### 5.1.2 Diesel generator constraint

The power generated from the diesel generators must be within the upper and lower limits of each generator.

$$P_{d.gen,i}^{\min}\leq P_{d.gen,i}(t)\leq P_{d.gen,i}^{\max}$$ (21)

#### 5.1.3 Power balance constraint

The primary constraint in the power system is the balance of demand and supply. The microgrid must balance the power flow each time step expressed by Eq (22)

$$P_{PV,out}(t)+P_{w,h}(t)+P_{batt}(t)+P_{d.gen}(t)-P_{L}(t)=0$$ (22)

## 6. Proposed method

The proposed method calculates the optimal battery size and performs economic scheduling of the distributed generators as per load demand at each hour. The economic scheduling is based on the power management strategies discussed above and is similar to the conventional method when there is an excess of energy through the renewable sources than that required by loads such as in scenarios 1 and 2 of the power management strategies. However, when the load power is greater than the renewable energy generation, the proposed algorithm will dispatch the power from diesel generators or the energy storage. The decision is based on the depth of discharge of the energy storage, which affects the operational cost of storage. The algorithm reads the DOD value at the start of the interval and then transforms the cost function given in Eq (7) from 3-dimensional to 2-dimensional plot.

**Fig 3:**
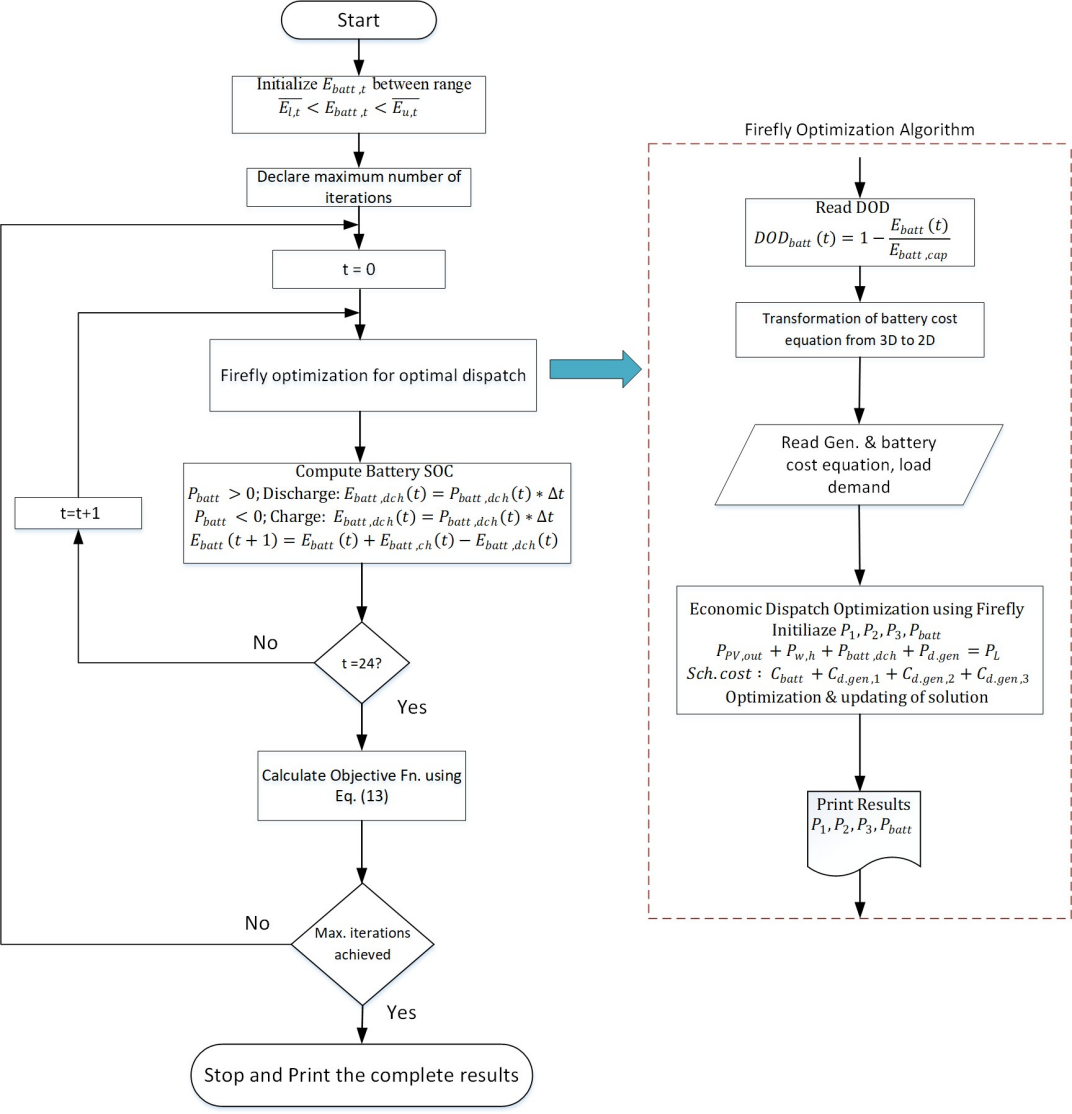
Flow chart of the proposed method.

The proposed optimization algorithm dispatches the optimal power from the three diesel generators and the energy storage, depending on the cost equations of the respective distributed power resources and the load demand at the specific hour. In addition, the proposed method also directs the diesel generators to charge the energy storage when the cost of the BESS per unit of energy is higher than the generator cost. At this stage, the battery DOD is high which may account for the power supply loss during the hours when the battery is required to dispatch power. The battery SOC is computed at the end of each hour after the algorithm takes the decision to charge or discharge the battery. The battery optimal size is calculated for the defined strategies so that the cost is minimum for the scheduling of the power resources which will reduce the overall cost of the microgrid. The battery optimal size is selected from a range such that the lower value $\bar{E_{l,t}}$ corresponds to the battery size in which there is no mismatch between the generation and load whereas the upper value $\bar{E_{u,t}}$ sustains the maximum charge at each interval. The battery size range in this paper is taken from 100kWh to 250 kWh. The flowchart of the proposed method is shown in Fig 3.

## 7. Firefly algorithm

The firefly algorithm (FA) analyzes the social behavior of flies and is similar to other meta-heuristic techniques. The algorithm was originally developed by Yang [32] based on three main ideas:

1. The fireflies attract their mating partners.
2. The bright firefly gets attracted towards brighter fireflies.
3. If the firefly cannot find brighter fireflies, then it will move randomly in the search space.

Like other optimization techniques such as artificial bee colony (ABC), PSO and harmony search algorithm (HSA), which are based on the population of search space, FA is also a population-based optimization algorithm. However, FA is distinguished from the other optimization techniques by adjusting the parameters, which have low dependency on the algorithm and appropriately identifying the search space. The above mentioned three ideas of firefly algorithm are explained in the mathematical form below:

### 7.1 Separation between fireflies

The distance between two mating fireflies in the search space is calculated as vector operation performed in Cartesian framework between *i*^th^ and *j*^th^ firefly given by the expression:

$$r_{ij}=\left|Y_{i}-Y_{j}\right|=\sqrt{\sum_{D=1}^{s}{\left(Y_{i,D}-Y_{j,D}\right)}^{2}}$$ (23)

where *r* is the distance between two fireflies, *S* is the dimension of control vector, *Y*_*i*,*D*_/*Y*_*j*,*D*_ are the D^th^ dimensions of *Y*_*i*_/*Y*_*j*_ fireflies respectively.

### 7.2 Attraction between firefly

The attraction of the fireflies decreases when the two mating fireflies moves in opposite direction and the separation between them increases. The attraction of the flies can be represented by the following expression:

$$\beta (r)=\beta_{0}\times \exp(-\gamma r^{m});\;m\geq 1$$ (24)

where *β*(*r*) and *β*_0_ represents the attractiveness when the fireflies are at the distance *r* and 0. *γ* is the coefficient of light absorbed by firefly and *m* is the number of fireflies taken as 2.

### 7.3 Movement of the fireflies

The fireflies move towards brighter fireflies. The movement between the two fireflies, *j*^th^ firefly (low intensity) towards the *i*^th^ firefly (high intensity) is given by mathematical expression:

$$Y_{j}(t)=Y_{j}+\beta_{0}\times \exp(-\gamma r^{m})\times (Y_{i}-Y_{j})+v_{j}$$ (25)

$$v_{j}=\delta (rand-0.5)$$ (26)

**Fig 4:**
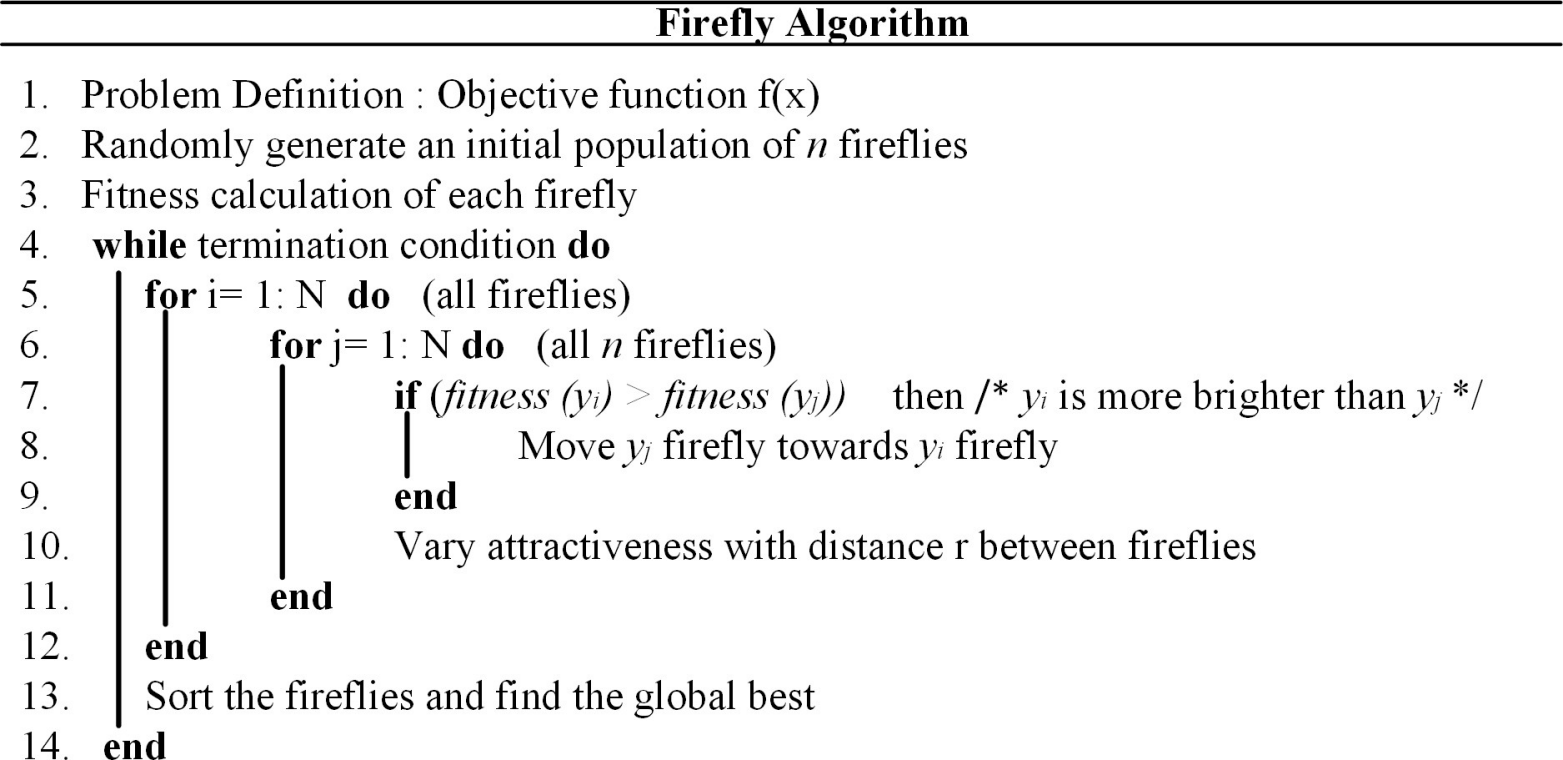
Pseudo code of firefly algorithm.

The first term of the Eq (25) shows the present position of *j*^th^ firefly. The second term represents intensity of brightness by which the *j*^th^ firefly is attracted towards *i*^th^ firefly. However, the last term *v*_*j*_ represents the movement of *j*^th^ firefly in the entire search space when it cannot find fireflies with more intensity. The randomization parameter *δ* is a constant value in the range of 0–0.5. The pseudo code of the firefly algorithm is shown in Fig 4.

## 8. Results and discussion

A typical low voltage microgrid with three diesel generators and a lithium-ion battery is analyzed in this study to illustrate the performance of the proposed energy management strategies. The microgrid consists of 68 kW photovoltaic and 37 kW wind turbine system. The data for the diesel generators are taken from [33] and is shown in Table 2. The battery capital cost, maintenance cost, interest rate and lifetime had been taken from [34]. The parameters for the wind turbine, solar photovoltaic, energy storage and optimization algorithm are represented in Tables 3–6 respectively. The proposed method is formulated in MATLAB (R2016b) and run on the personal computer 2.6 GHz core i5 processor with 6 GB RAM. The computational time involved in the simulation is about 3 min 15 sec.

**Table 2:** Parameters of diesel generators.

DG	*a*_*i*_ ($/kW^2^)	*b*_*i*_ ($/kW)	*c*_*i*_ ($)	*P*_*min*_ (kW)	*P*_*max*_ (kW)
Diesel 1	0.0001	0.0438	0.3	0	40
Diesel 2	0.0001	0.0479	0.5	0	20
Diesel 3	0.0001	0.0490	0.4	0	10

**Table 3:** Parameters of wind turbine.

Component Parameter	Value
Rated Power (kW)	37
Cut-in speed (m/s)	2.5
Cut-out Speed (m/s)	16
Rated speed (m/s)	7
Initial Capital cost ($/kW)	2000
Lifetime (year)	10
Interest rate (%)	6

**Table 4:** Parameters of solar PV.

Component Parameter	Value
Rated Power (kW)	68
Initial Capital cost ($/kW)	3000
Lifetime (year)	10
Interest rate (%)	6

**Table 5:** Parameters of energy storage.

Component Parameter	Value
Initial SOC (%)	75
$SOC_{batt}^{\max}$ (%)	90
$SOC_{batt}^{\min}$ (%)	15
Initial capital cost ($/kWh)	625
Maintenance cost ($/kWh)/year	25
Round-trip Efficiency (%)	90
Lifetime (years)	3
$P_{batt}^{min}$ (kW)	10
$P_{batt}^{max}$ (kW)	25
Interest rate (%)	6

**Table 6:** Parameters of optimization algorithm.

Parameter	Value
Size of population	150
Number of iterations	1000
*β*_0_	2
*δ*	0.2
*γ*	1
*m*	2

**Fig 5:**
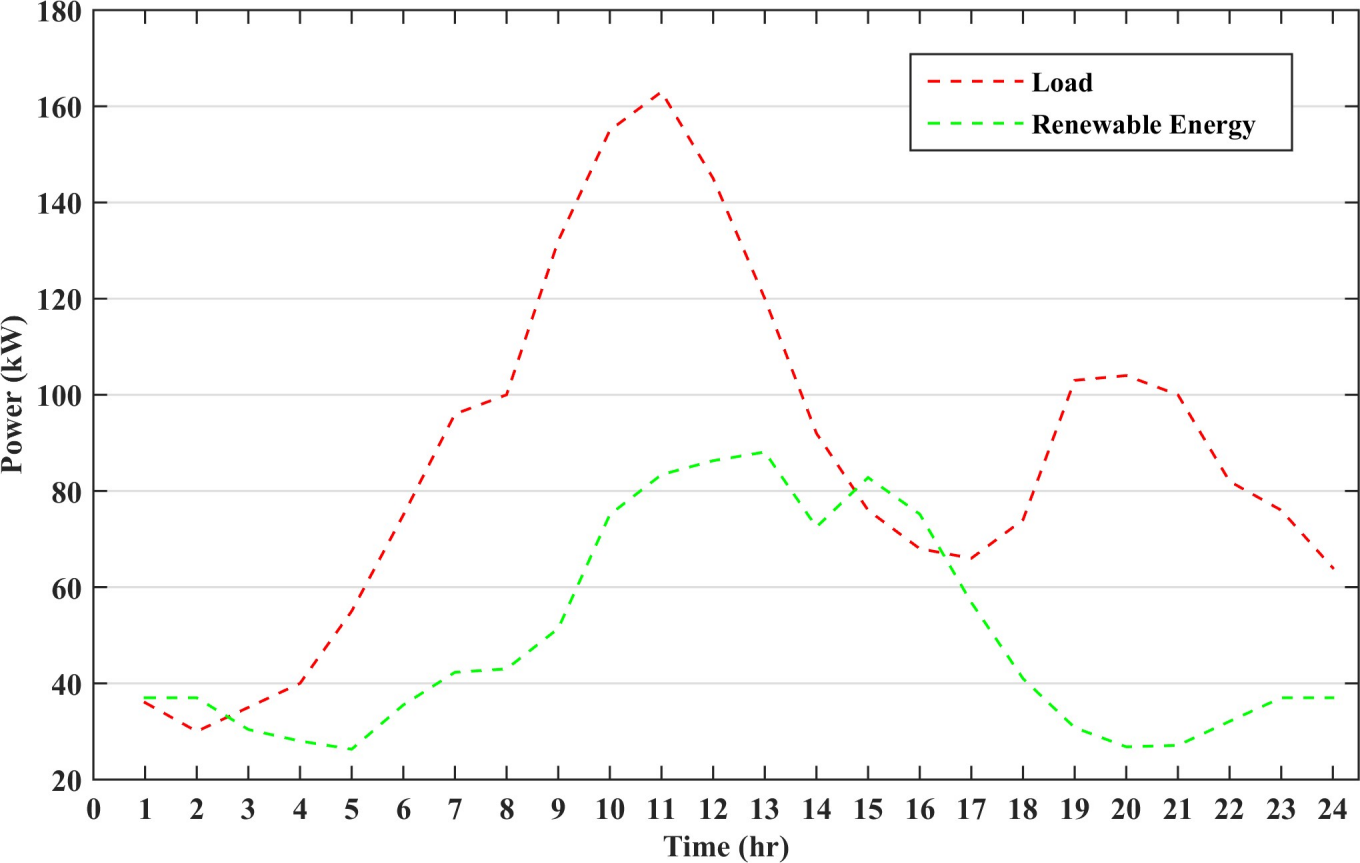
Renewable energy and load data for a day.

The generation subsystem is designed to meet the peak load. However, intermittency of RESs and consumers’ behaviors may affect the electricity cost and system reliability. A typical load profile of small residential area with the peak load of 163 kW has been taken in this study. The microgrid load profile and renewable energy generation graphs are shown in Fig 5. The load demand at most of the instances are higher than the combined wind and solar power generation. Thus, the generator and battery storage will be operated at these instances. However, there are certain instances when the renewable power generation is slightly higher than the load demand. Hence, the surplus power will charge the energy storage and the generators remains in the rest state during these time intervals. The diesel generator will charge the battery storage when the cost of battery storage becomes higher than the diesel generator cost. This increase in BESS cost is due to the continuous discharge of battery, which raises its DOD value. The battery must also be charged to avoid any load shedding or power failure in the next hours due to uncertain nature of RESs. This will increase the lifetime of the battery as the battery will operate at lower DOD values. The battery storage used in the microgrid operation is assumed to be initially charged at 75% SOC.

The proposed strategy is implemented based on the three cases below and the operating cost is computed in each case.

### 8.1 Case A: System is running without the battery storage

**Fig 6:**
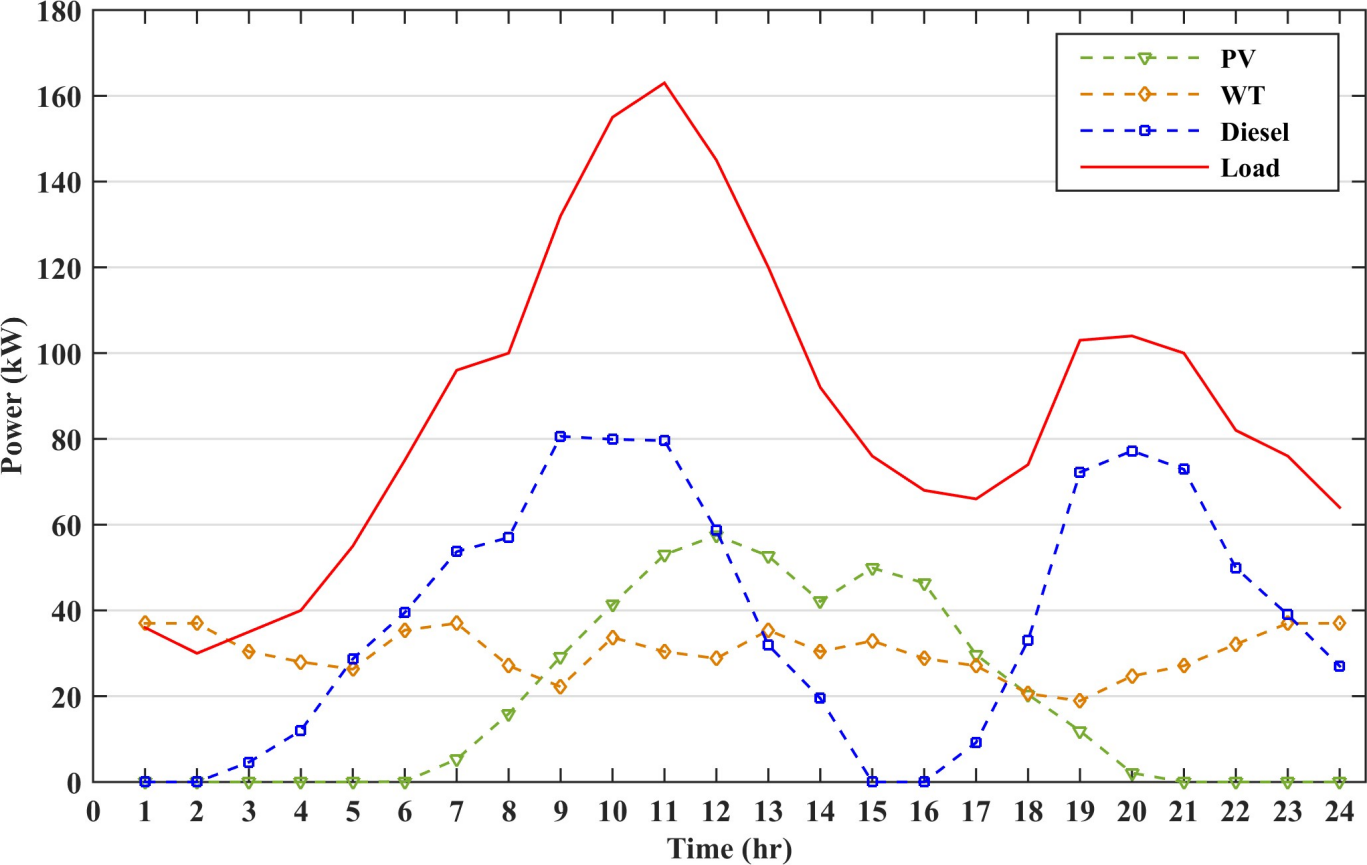
Microgrid operation without the battery storage.

The microgrid is operated without the energy storage. Thus, there will be no cost for the TCPD and the objective function is restricted to the daily economic scheduling of the microgrid. The RESs and diesel generators have to satisfy the load demand at all instances. However, there are some instances where the renewable and diesel generators cannot fulfil the required load, thus, there will be power mismatch between the generation and demand. This power mismatch will imbalance the system and will result in a power loss due to load shedding. The load shedding accounts for the penalty to be imposed increasing the scheduling cost of the microgrid. However, when the RES power is higher than the loads, the surplus power from the renewable energy sources will be dissipated as a dump load. Fig 6 shows the generation curve of the distributed sources and the load profile.

### 8.2 Case B: Battery size of 100 kWh is added to system

**Fig 7:**
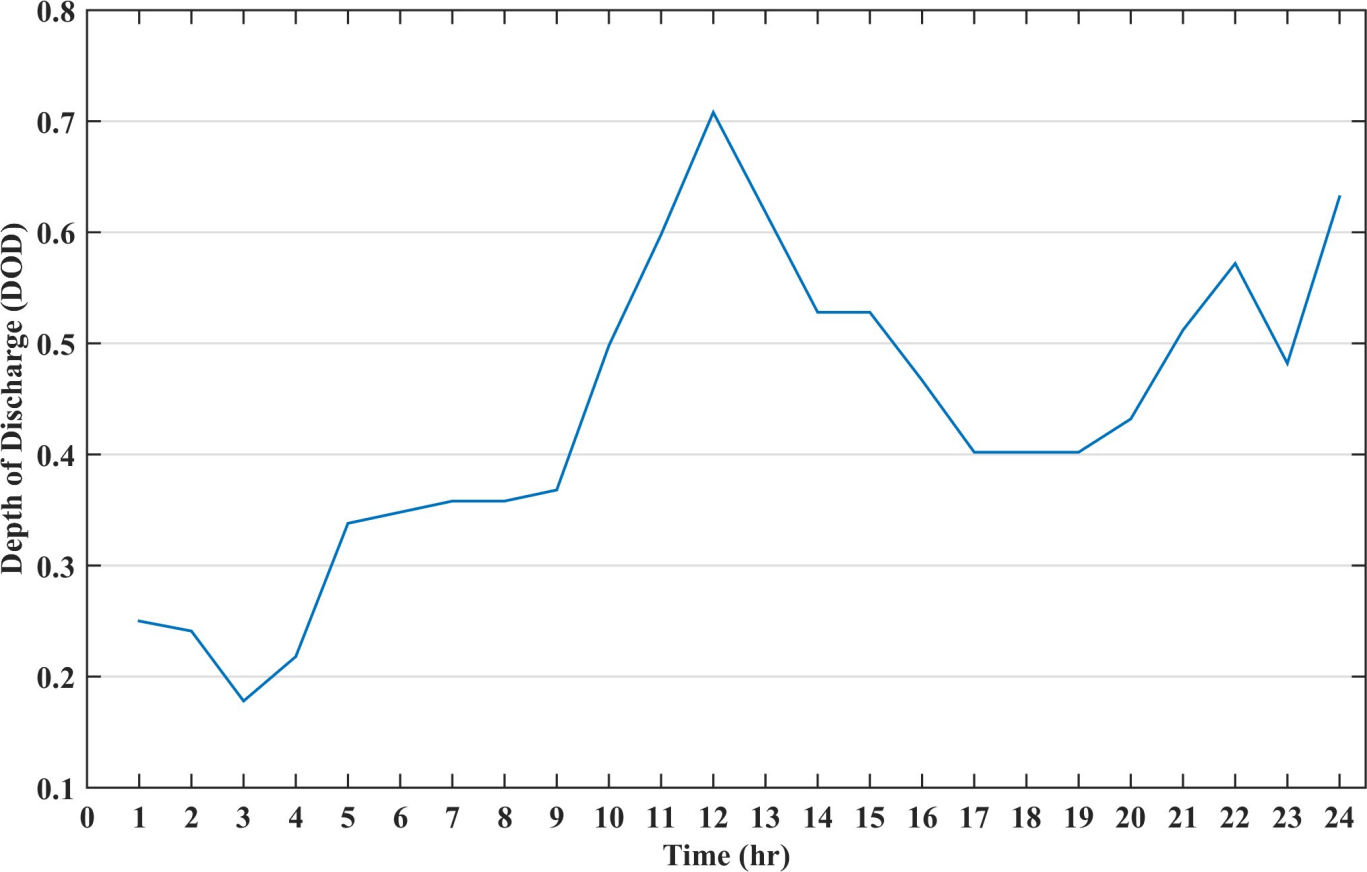
The depth of discharge status of battery for case B.

**Fig 8:**
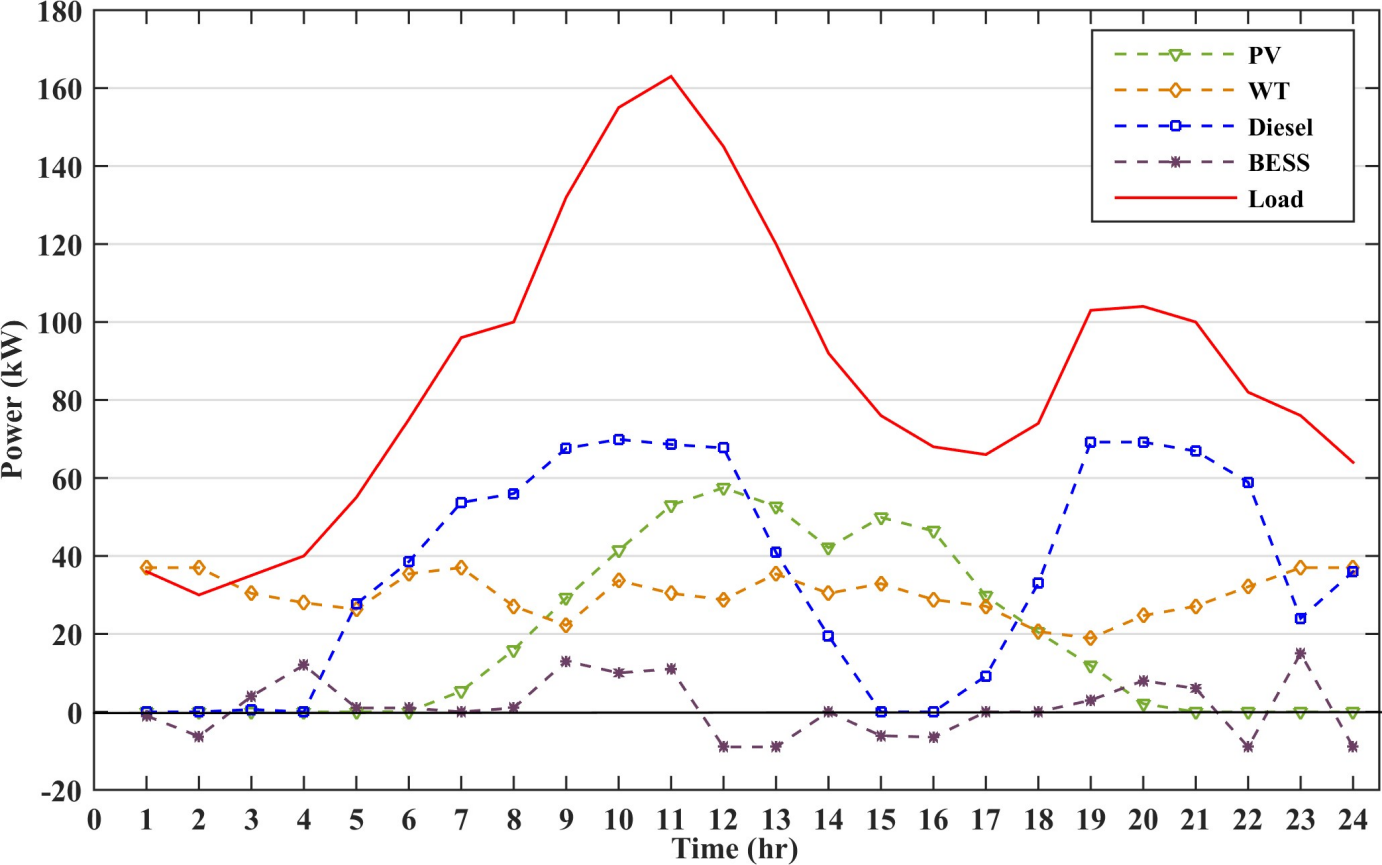
Operation of microgrid with all power generations and load for battery size of 100kWh.

The renewable sources and diesel generator cannot meet the load demand at all instances, hence, the battery storage must be installed in the microgrid system. In this case, a battery size of 100 kWh is added to the system. The battery size is selected such that there is no mismatch between the generation and the load at any time of the day. The battery is discharged when the cost of battery is lower than generator cost and charged when the battery cost becomes higher. The scheduling cost of the microgrid for the day in this case is calculated as $281.37 whereas the battery TCPD is $ 70.90. The cost of electricity of a day for this load profile is 18.44 (cents/kWh). Fig 7 shows the battery DOD for this case and it can be seen from the figure that during the certain instance, the DOD becomes higher than 70%, which will greatly impact the cost of energy storage during charge/discharge. Also, the battery charge at the end of the day is low which may impact the operation of the next day. The operation of all the distributed resources in the microgrid with battery size of 100 kWh is represented in Fig 8. In this figure, diesel is the accumulative sum of all the three diesel generators power dispatch.

### 8.3 Case C: Optimal battery size is added to the system

**Fig 9:**
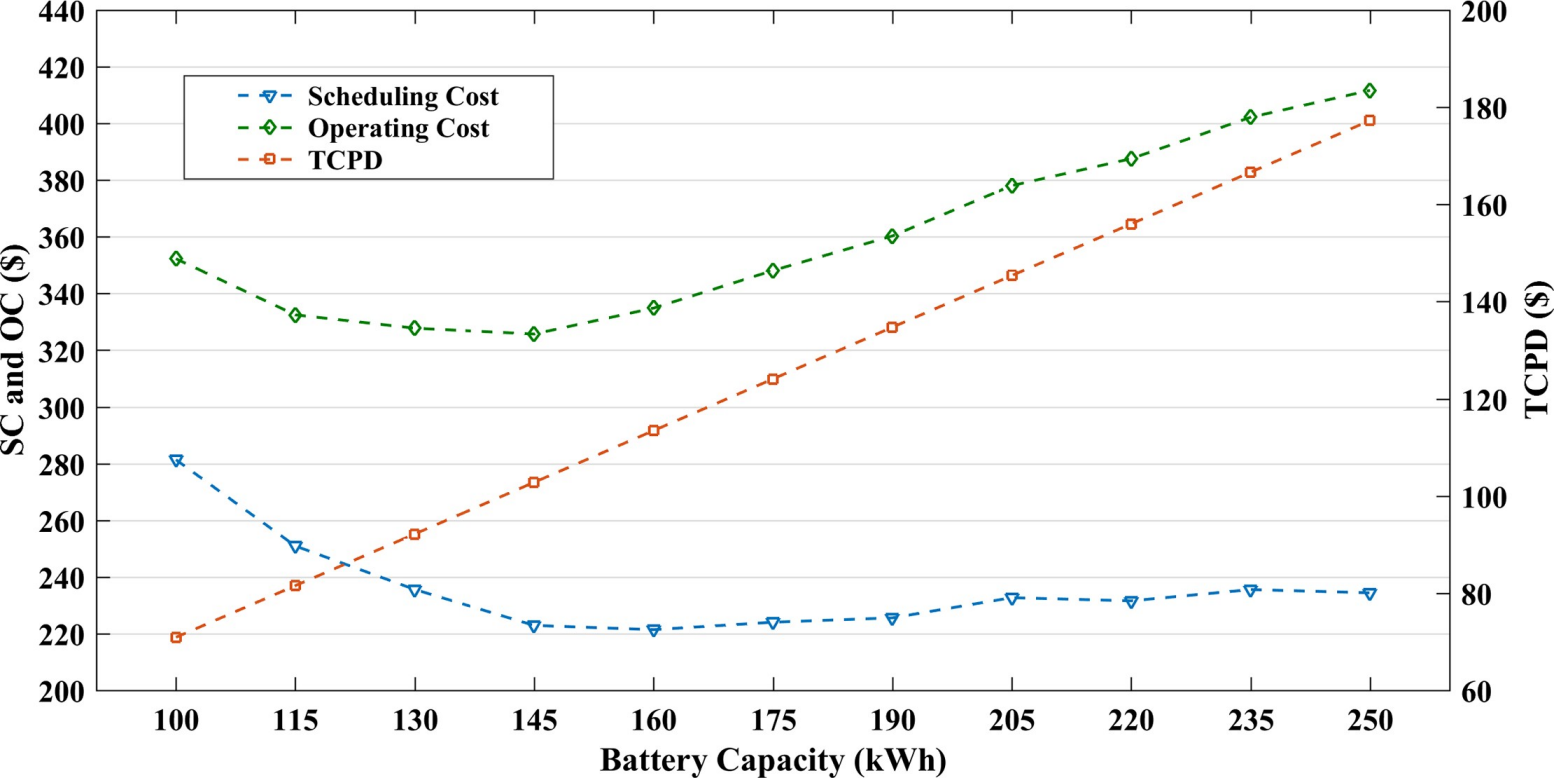
Microgrid operating cost for different battery sizes.

The optimal battery size for the microgrid operation is determined to produce a cost-effective system. The proposed algorithm computes the optimal battery size to minimize the OC of microgrid. The proposed method calculates the battery size to be 145.5 kWh. The operating cost for the optimal size is found to be $ 325.62. To validate the result of the proposed method, the microgrid OC has been computed for the battery size within the range of 100kWh - 250kWh with a step size of 15kWh, considering all the constraints of the distributed energy resources and the battery. Fig 9 shows the scheduling cost, TCPD and the overall OC for the different battery sizes. The optimum battery capacity for this system is recorded to be 145kWh, similar to that by the proposed method. The results show that the scheduling cost is high for smaller battery sizes, and as the size is increased the cost slightly reduces. This trend is followed until the battery size reaches up to 145kWh after which the scheduling cost increases again. The TCPD of battery is a linear curve which increases with the size of the battery. The OC for the microgrid shows a very small change in the cost until the battery size reaches 145kWh and gradually increases thereafter.

**Fig 10:**
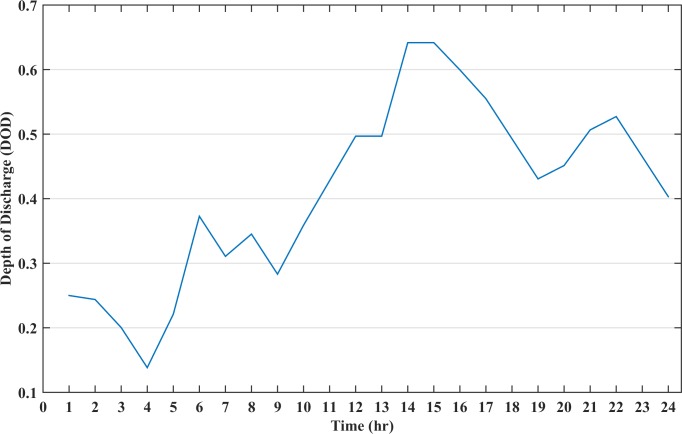
The depth of discharge status of battery for case C.

The battery DOD for the entire time period is shown in Fig 10. It can be seen from the figure that during the initial hours, the battery is discharging with an increasing DOD value. At 06:00 the battery DOD has raised to 38% at which level it upsurges the battery cost while the generator discharges more power and simultaneously charges the battery. After 09:00, the generator alone cannot fulfil the load demand, hence, the battery has to discharge in these instances to avoid load shedding. The DOD value at these instances increases and the battery discharges irrespective of the high cost. As soon as the high load period ends, the generator charges the battery again, to ensure sufficient charge during the critical hours. The critical hours are considered as those hours when the renewable energy and diesel generator working together cannot meet the load demand; despite the high cost of the battery storage, it discharges power to fulfil the load demand. Thus, the battery is never depleted completely and avoids deep discharges which prolongs the battery lifetime.

**Fig 11:**
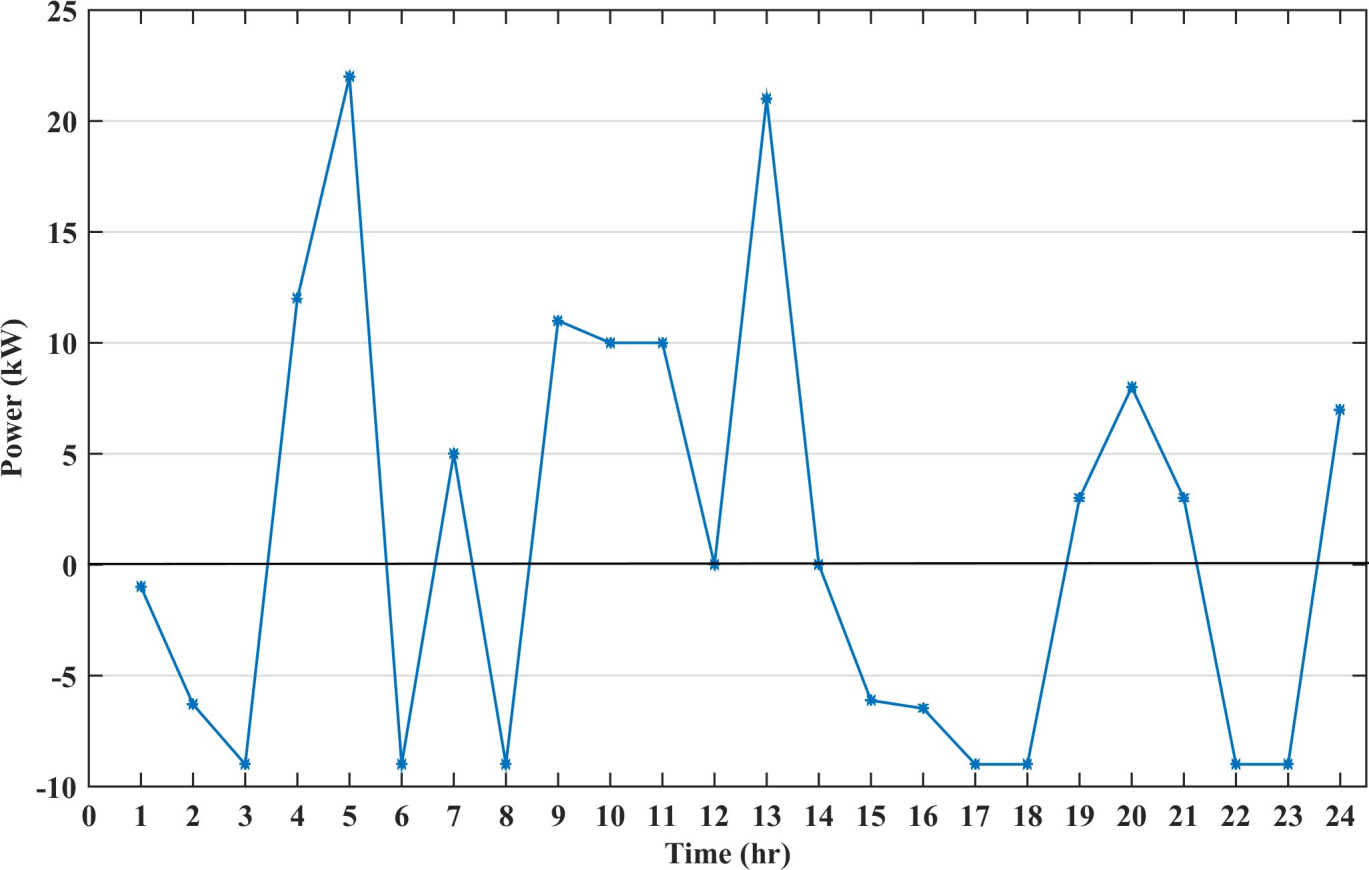
The battery charging and discharging power analysis.

The battery charging and discharging power analysis is shown in Fig 11. The battery discharging power is represented by a positive value whereas the negative value shows the charging process. The figure shows that most of the time the battery is discharging. The discharged power varies during these instants. The maximum discharge power in a one-time sequence is set to 25kW. This is to avoid complete discharge of the battery storage in one interval so that the battery can be utilized during critical hours. The renewable power generation is high during the hours 00:00–02:00 and 15:00–17:00 and the excess energy is stored in the battery.

**Fig 12:**
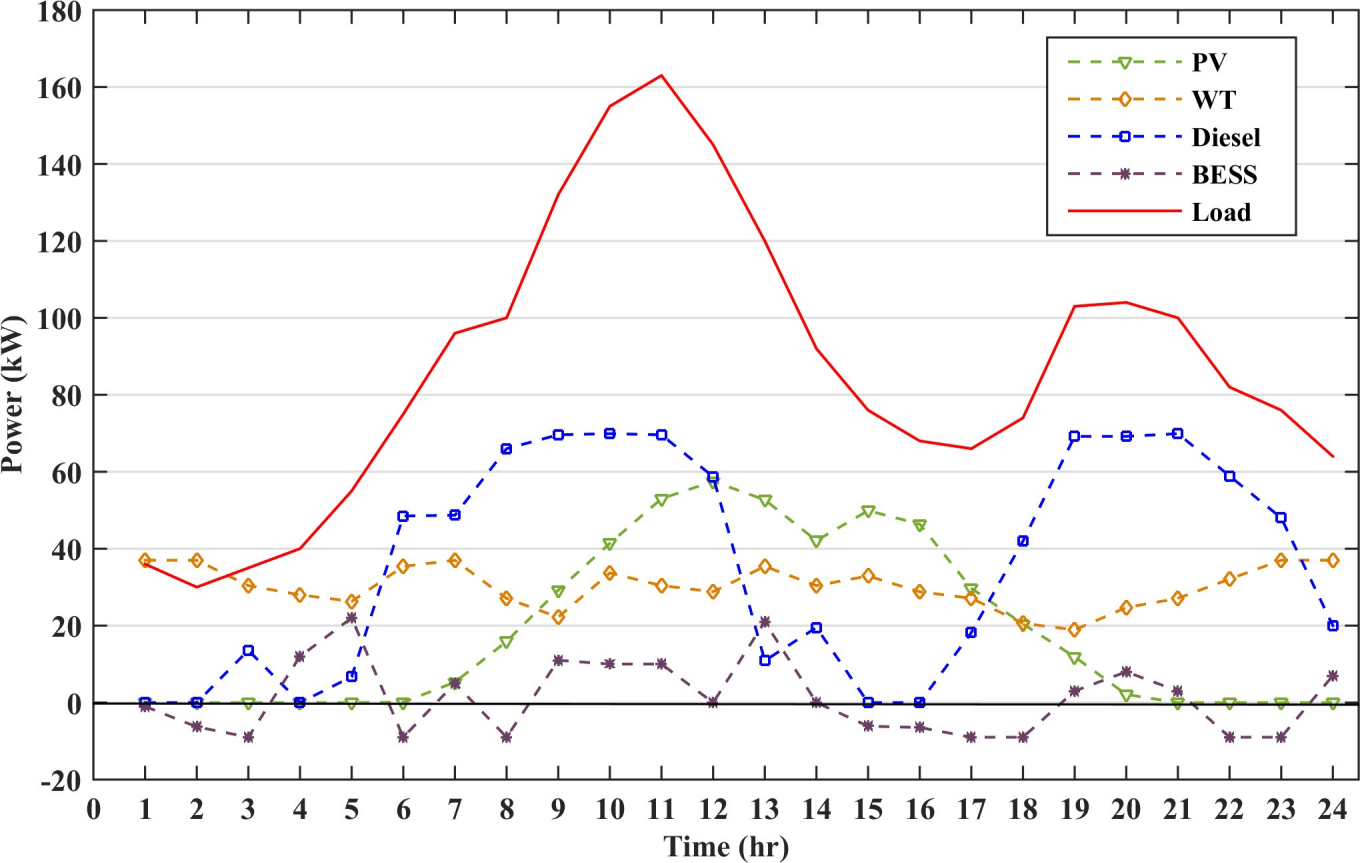
Operation of microgrid with all generations and load.

The generation from all the distributed resources and the load demand are plotted in Fig 12. Diesel generator 1 has the lowest cost among the other diesel generators and it also dispatches maximum power. Generator 3 is given the least priority among the three generators due to its high cost. The generation and load are balanced at all the instances which restricts any penalty to be imposed thus reducing the overall operating cost.

**Fig 13:**
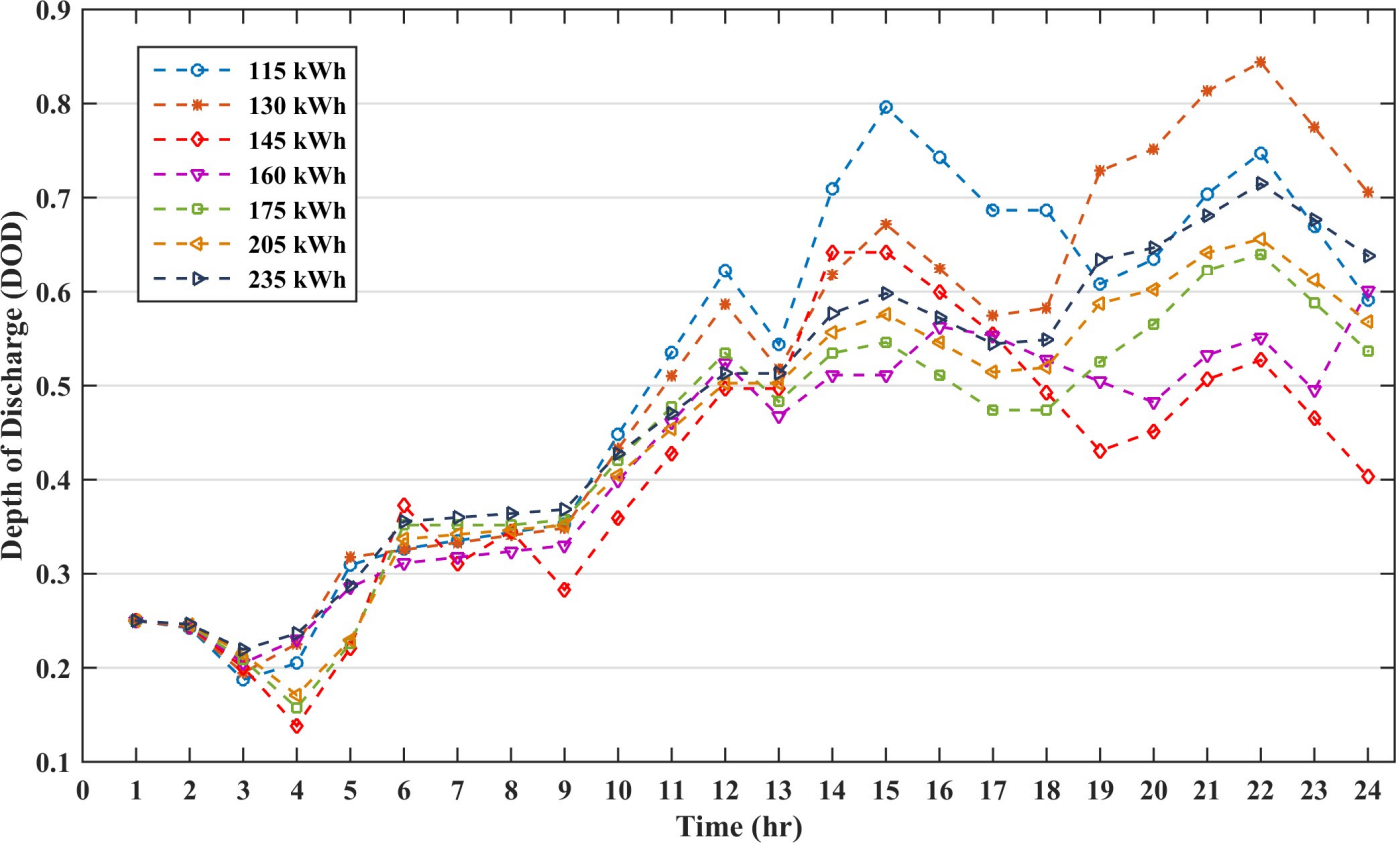
DOD curves of different battery sizes.

The results for the above cases are tabulated in Table 7. The renewable power generation in all the cases is identical, and the economic dispatch is performed for the power difference between the load and the renewable energy. The negative values in the BESS shows the charging schedule while the positive values represent the discharging schedule. Generator 1 dispatches more power due to it being the cheapest among the three generators, whereas generator 3 dispatches minimal power. The battery DOD curves for the different battery sizes are shown in Fig 13. The figure shows that DOD values for the optimal battery size of 145 kWh is lowest at most of the instances as compared to other battery sizes. The lifecycle and lifespan of the different battery sizes are tabulated in Table 8 by taking the average of the DOD. The optimal battery capacity results in longer lifetime in comparison to other battery capacities and the conventional method [26]. Thus, the optimal size and the economic scheduling may prolong the battery lifetime and reduce the microgrid cost.

**Table 7:** Scheduling results of different cases at every hour.

Time (hr)	Solar PV	Wind	Load-RES	Economic dispatch without BESS				Economic dispatch with 100kWh Battery size				Economic dispatch with optimal battery size			
				G1	G2	G3	BESS	G1	G2	G3	BESS	G1	G2	G3	BESS
1	0	37	-1	0	0	0	0	0	0	0	-0.9	0	0	0	-0.9
2	0	37	-7	0	0	0	0	0	0	0	-6.3	0	0	0	-6.3
3	0	30.4	4.6	4.6	0	0	0	0	0	0	4.6	13.6	0	0	-9
4	0	28	12	12	0	0	0	0	0	0	12	0	0	0	12
5	0	26.3	28.7	24.7	4	0	0	24	3.7	0	1	6.7	0	0	22
6	0.1	35.4	39.5	28.7	8.1	2.7	0	28.3	7.8	2.4	0	31.6	11.2	5.7	-9
7	5.3	37	53.7	33.4	12.8	7.5	0	33.4	12.8	7.5	0	38.6	8.5	1.6	0
8	15.9	27.1	57	34.5	14	8.5	0	34.1	13.7	8.2	0	38.3	17.7	10	0
9	29.2	22.2	80.6	40	20	20.6	0	39.1	18.5	10	13	40	19.6	10	11
10	41.4	33.7	79.9	40	20	19.9	0	40	19.9	10	10	40	19.9	10	10
11	53	30.4	79.6	40	20	19.6	0	39.6	19	10	11	40	19.6	10	10
12	57.5	28.8	58.7	35	14.6	9.1	0	39.1	18.6	10	-9	39.9	11.7	7.1	0
13	52.7	35.4	31.9	26.1	5.6	0.2	0	29.1	8.6	3.2	-9	10.9	0	0	21
14	42.1	30.4	19.5	19.5	0	0	0	15.4	2.3	1.8	0	6.2	10.3	3	0
15	49.9	32.9	-6.8	0	0	0	0	0	0	0	-6.12	0	0	0	-6.12
16	46.4	28.8	-7.2	0	0	0	0	0	0	0	-6.48	0	0	0	-6.48
17	29.7	27.1	9.2	9.2	0	0	0	9.2	0	0	0	18.2	0	0	-9
18	20.4	20.6	33	26.5	6	0.5	0	26.9	6.1	0	0	29.5	9	3.5	-9
19	11.9	18.9	72.2	39.6	19	13.6	0	39.9	19.3	10	3	39.9	19.3	10	3
20	2.1	24.7	77.2	40	20	17.2	0	39.9	19.3	10	8	39.9	19.3	10	8
21	0	27.1	72.9	39.9	19.3	13.7	0	38.7	18.2	10	6	40	19.9	10	3
22	0	32.1	49.9	32.1	11.7	6.1	0	35.1	14.7	9.1	-9	35.1	14.7	9.1	-9
23	0	37	39	28.5	8	2.5	0	0	20	4	15	31.5	11	5.5	-9
24	0	37	27	23.8	3.2	0	0	20.5	7	1.5	-9	16.3	7.4	3.3	7

**Table 8:** Lifetime analysis for different battery capacities.

Battery capacities (kWh)	Average DOD value (%)	Lifecycles (cycle)	Lifetime (year)
115	51.15	1183	3.2
130	51.30	1180	3.2
145	40.08	1435	3.9
160	45.24	1303	3.5
175	43.47	1346	3.7
190	45.58	1296	3.6
205	44.71	1316	3.5
215	46.92	1266	3.5
235	47.67	1250	3.4
145 (method proposed in [26])	54.55	1123	3.0

### 8.4 Comparison of proposed technique with the conventional technique

**Fig 14:**
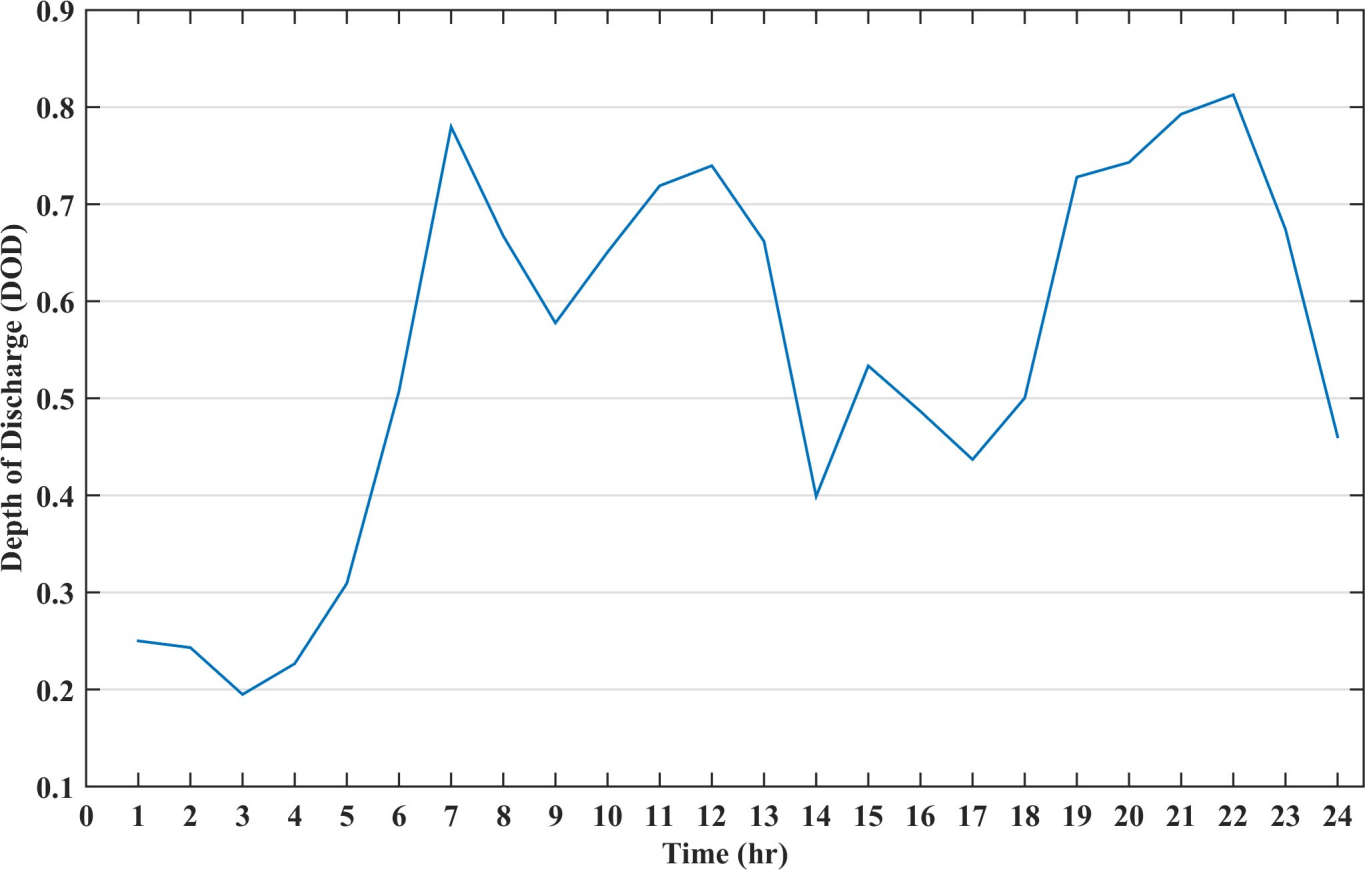
Battery depth of discharge status for the conventional method.

**Fig 15:**
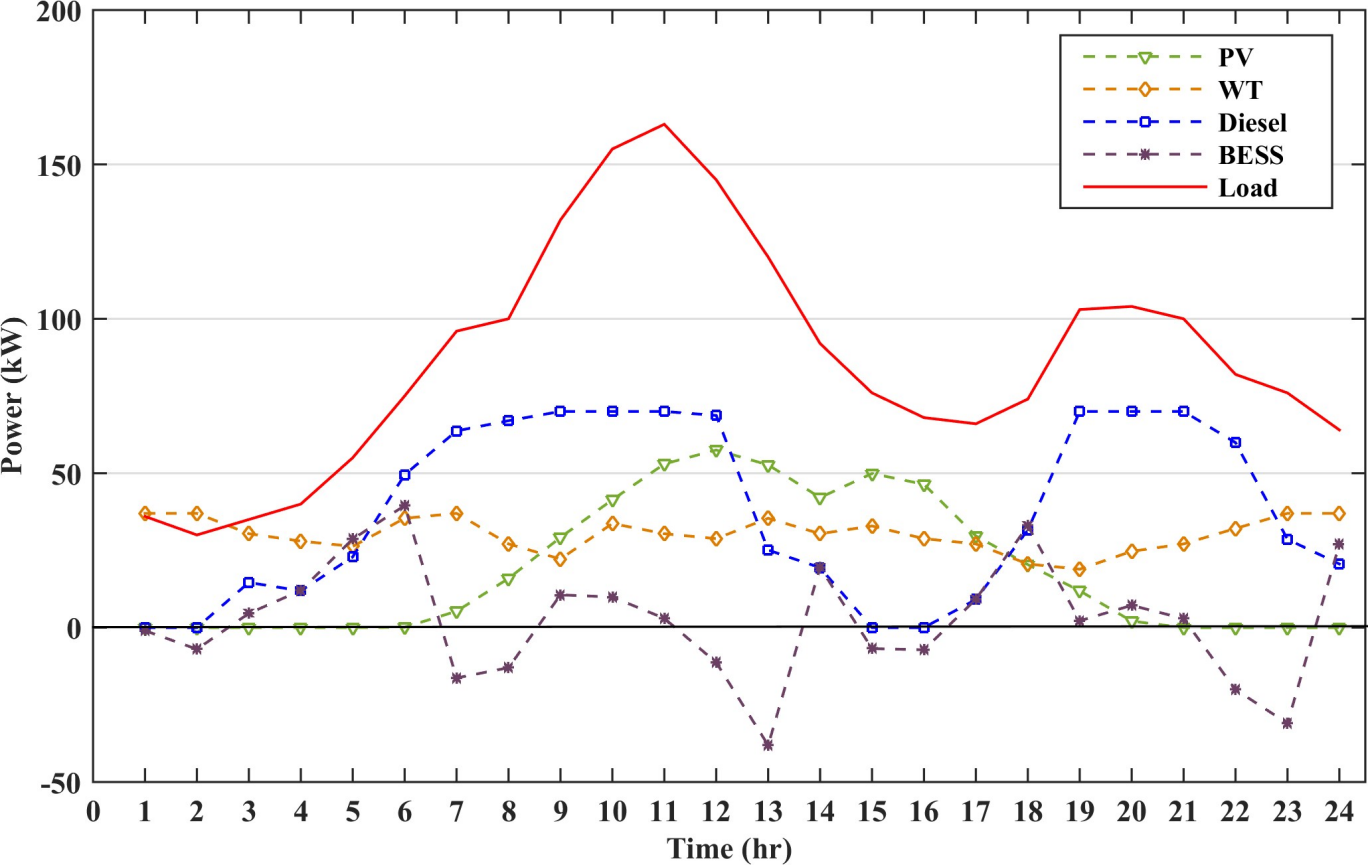
Microgrid operation with all the generations and load.

The effectiveness of the proposed method is verified by comparing it with other conventional methods [26, 35] in which the battery storage discharges when the renewable resources fail to provide sufficient energy to meet the load. When the battery reaches minimum energy level, the diesel generator is turned on to charge the battery storage. This method reduces the lifetime of battery storage by continuously discharging to the minimum level. The authors in [26] have used PSO to get the optimal power dispatch. The comparison is done by implementing the conventional method using the above-mentioned parameters, and the results are shown in Table 9. It is apparent from the table that the proposed method reduces the operating cost by 50% as compared to the conventional method. The battery depth of discharge status for the conventional method is shown in Fig 14 which depicts that the DOD is higher at most of the instances over 24 hours due to complete power discharge from the energy storage. Thus, the operating cost of the microgrid by using the conventional method is high as compared to the proposed method. The overall power generations and load for all the time intervals are plotted in Fig 15.

**Table 9:** Comparison of proposed method with the conventional method.

Method	Scheduling Cost ($)	Microgrid Operating Cost ($)	Average COE (cents/kWh)
Conventional method	557.09	659.90	31.65
Proposed Method	222.87	325.68	15.63

### 8.5 Comparison of proposed optimization algorithm with other algorithms

**Fig 16:**
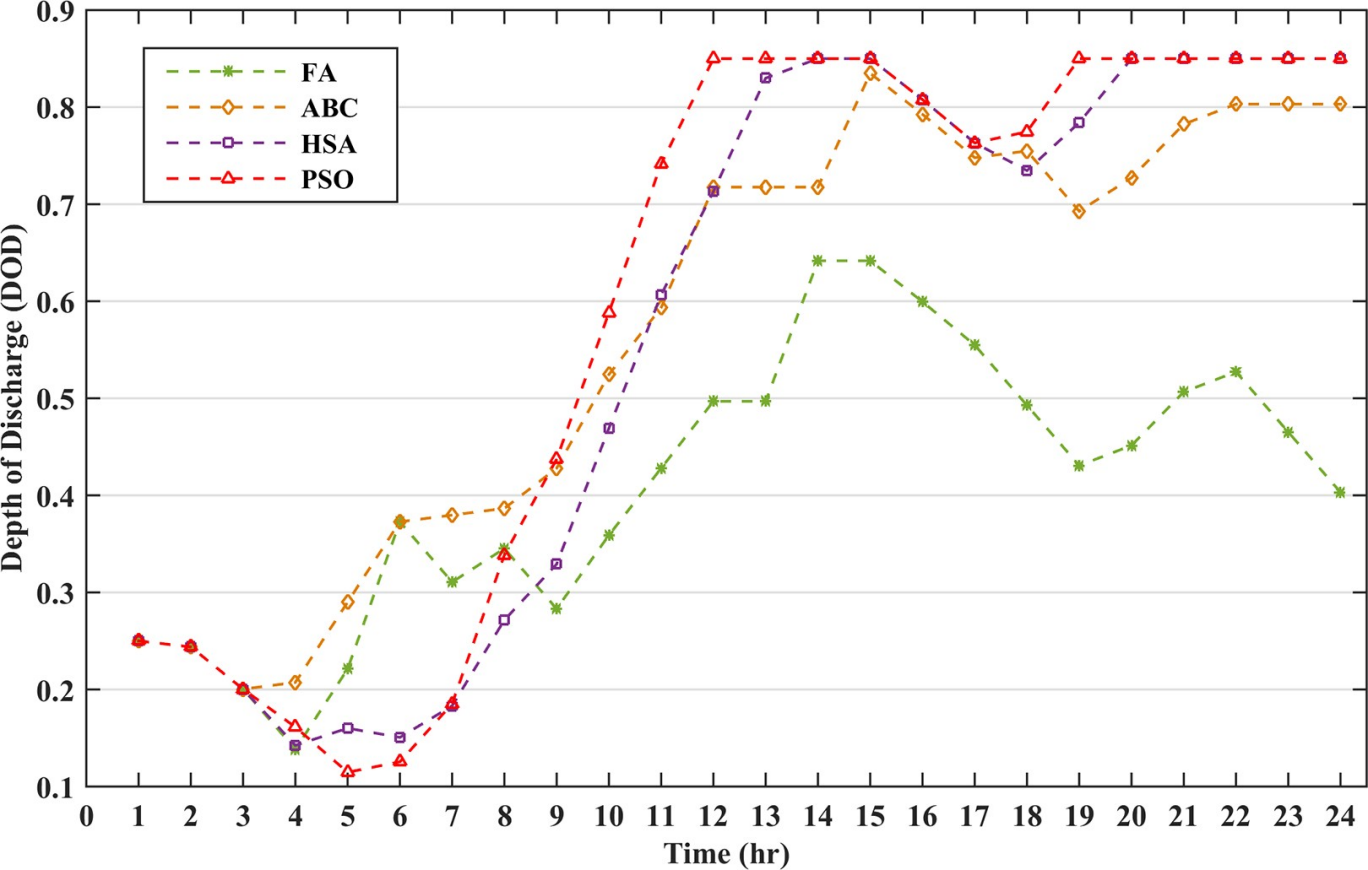
Depth of discharge status.

**Fig 17:**
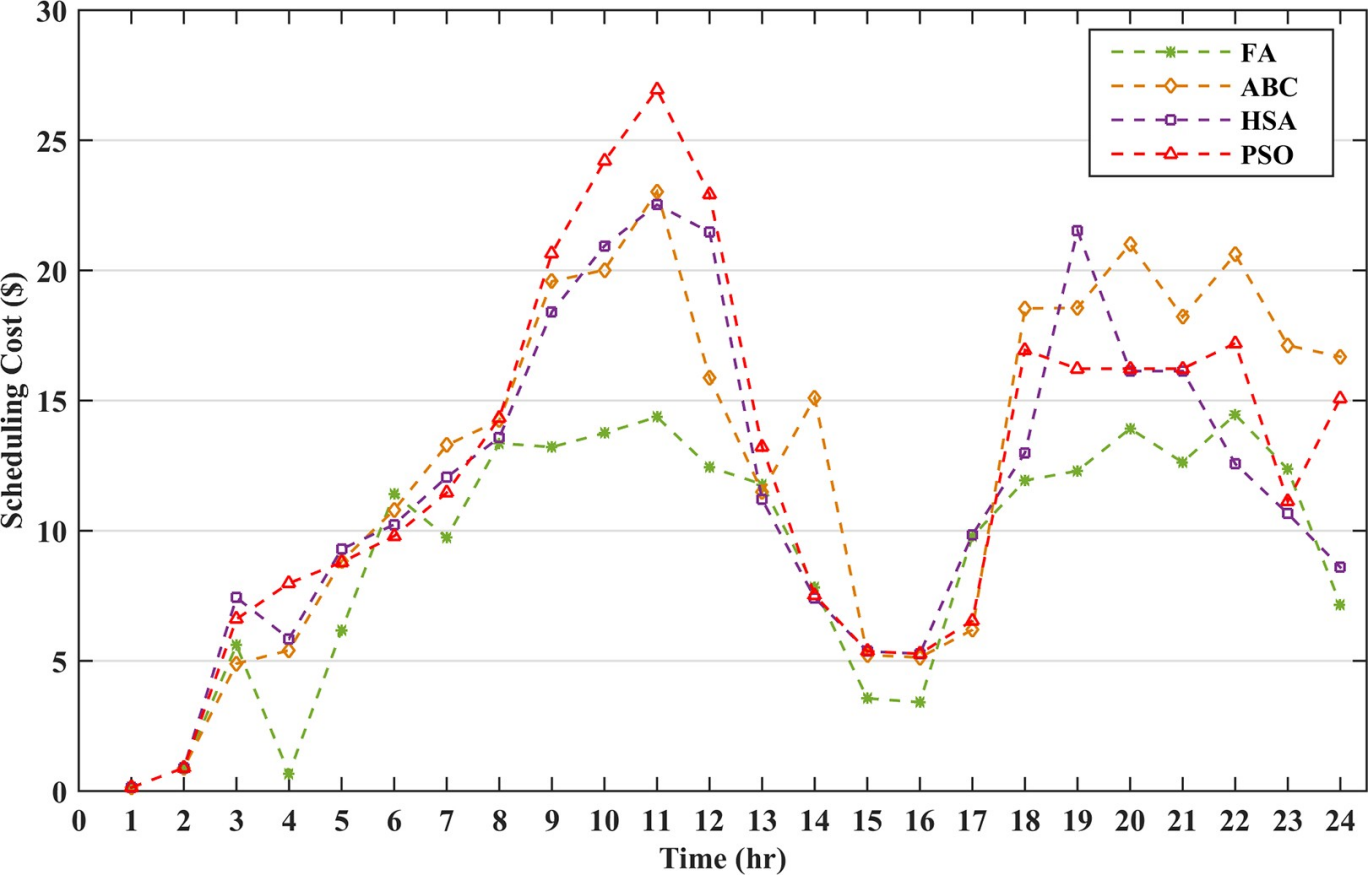
Hourly scheduling cost.

The robustness of the FA is analyzed by implementing ABC, HSA and PSO for the proposed method and the results are reported in Table 10. The table shows that FA has the minimum operating cost with 0% LPSP. However, PSO and HSA are not capable to meet the load demand at all the instances resulting in the load shedding and has a higher LPSP ratio. The battery discharging cost is the ratio of the accumulated battery cost at all the instances in which the BESS is discharged to the sum of the power discharged by the BESS. The battery discharging cost of FA is the minimum of all the other algorithms and depicts that the BESS does not discharge more power at high DOD values thus reducing the scheduling cost of the microgrid. The battery DOD has been compared for all the algorithms and the results are shown in Fig 16. The results illustrate that FA has been capable of limiting the battery DOD to a low value to minimize the battery operational cost. The scheduling cost for the hourly analysis with the battery size of 145kWh had been compared with the above-mentioned algorithms as in Fig 17. The figure clearly depicts that the cost at each hour by FA is comparatively lower than other algorithms which reduces the overall operating cost of the microgrid.

**Table 10:** Comparison of different algorithms for the proposed method.

Method	Microgrid Operating Cost ($)	Average COE (cents/kWh)	Average Battery Discharging Cost (cents/kWh)	LPSP (%)	Computational time (sec)
Artificial Bee Colony	393.10	18.86	39.24	0	265
Harmony Search Algorithm	383.34	18.40	37.00	37.5	225
Particle Swarm Optimization	404.46	19.41	38.02	25	250
Firefly Algorithm	325.68	15.63	22.13	0	195

## 9. Conclusion

As more energy supplies are predicted to utilize renewable sources, the economic and battery sizing aspects of the energy storage in the isolated microgrid has to be taken into consideration to ensure a reliable service. The present study has solved the economic scheduling problem between the diesel generators and the battery storage considering real time battery degradation cost. One of the strengths of the proposed method is to charge the battery when the DOD value is high so that the battery is not depleted during critical hours. Firefly optimization algorithm was implemented to solve the economic dispatch and the battery sizing problem. The simulation results reveal that the microgrid faces the load shedding without the battery storage resulting in high operating cost and instability. Furthermore, a large BESS size does not minimize the operating cost, but there exists an optimal point, which should be considered when designing a microgrid system. The lifespan of the battery is also extended when optimal size is selected for economic scheduling saving cost of replacing BESS. The proposed method has been compared with other existing methods and 50% reduction in operating cost has been recorded. Thus, the obtained results show that ignoring the depth of discharge and lifetime of the BESS in an economic scheduling will inflate the operating cost of the microgrid. The energy scheduling approach presented will help the independent power plant operators to perform the rural electrification efficiently and prolong the battery lifetime.

## Supporting information

S1 File Microgrid data. (PDF)

## References

[1] M. Jamshidi and A. Askarzadeh, "Techno-economic analysis and size optimization of an off-grid hybrid photovoltaic, fuel cell and diesel generator system," Sustainable Cities and Society, 2018/10/15/ 2018.

[2] H. Kuang, S. Li, and Z. Wu, "Discussion on advantages and disadvantages of distributed generation connected to the grid," in Electrical and Control Engineering (ICECE), 2011 International Conference on, 2011, pp. 170–173.

[3] Alsaidan I., Khodaei A., and Gao W., "A Comprehensive Battery Energy Storage Optimal Sizing Model for Microgrid Applications," *IEEE Transactions on Power Systems*, pp. 1–1, 2017.

[4] Robert F. C., Sisodia G. S., and Gopalan S., "A critical review on the utilization of storage and demand response for the implementation of renewable energy microgrids," *Sustainable Cities and Society*, vol. 40, pp. 735–745, 2018/07/01/ 2018.

[5] Carta J. A. and Velázquez S., "A new probabilistic method to estimate the long-term wind speed characteristics at a potential wind energy conversion site," *Energy*, vol. 36, pp. 2671–2685, 2011.

[6] Castronuovo E. D. and Lopes J. P., "On the optimization of the daily operation of a wind-hydro power plant," *IEEE Transactions on Power Systems*, vol. 19, pp. 1599–1606, 2004.

[7] Eyer J. and Corey G., "Energy storage for the electricity grid: Benefits and market potential assessment guide," *Sandia National Laboratories*, vol. 20, p. 5, 2010.

[8] Fu Q., Hamidi A., Nasiri A., Bhavaraju V., Krstic S. B., and Theisen P., "The Role of Energy Storage in a Microgrid Concept: Examining the opportunities and promise of microgrids," *IEEE Electrification Magazine*, vol. 1, pp. 21–29, 2013.

[9] Aghamohammadi M. R. and Abdolahinia H., "A new approach for optimal sizing of battery energy storage system for primary frequency control of islanded microgrid," International Journal of Electrical Power & Energy Systems, vol. 54, pp. 325–333, 2014.

[10] T. Kerdphol, Y. Qudaih, and Y. Mitani, "Battery energy storage system size optimization in microgrid using particle swarm optimization," in Innovative Smart Grid Technologies Conference Europe (ISGT-Europe), 2014 IEEE PES, 2014, pp. 1–6.

[11] Khorramdel H., Aghaei J., Khorramdel B., and Siano P., "Optimal Battery Sizing in Microgrids Using Probabilistic Unit Commitment," *IEEE Trans*. *Industrial Informatics*, vol. 12, pp. 834–843, 2016.

[12] Mehmood K. K., Khan S. U., Lee S.-J., Haider Z. M., Rafique M. K., and Kim C.-H., "Optimal sizing and allocation of battery energy storage systems with wind and solar power DGs in a distribution network for voltage regulation considering the lifespan of batteries," *IET Renewable Power Generation*, vol. 11, pp. 1305–1315, 2017.

[13] Bahramirad S., Reder W., and Khodaei A., "Reliability-Constrained Optimal Sizing of Energy Storage System in a Microgrid," *IEEE Transactions on Smart Grid*, vol. 3, pp. 2056–2062, 2012.

[14] Rokni S. G. M., Radmehr M., and Zakariazadeh A., "Optimum energy resource scheduling in a microgrid using a distributed algorithm framework," *Sustainable Cities and Society*, vol. 37, pp. 222–231, 2018/02/01/ 2018.

[15] Nojavan S., Majidi M., and Esfetanaj N. N., "An efficient cost-reliability optimization model for optimal siting and sizing of energy storage system in a microgrid in the presence of responsible load management," *Energy*, vol. 139, pp. 89–97, 2017.

[16] Nguyen T. A., Crow M. L., and Elmore A. C., "Optimal sizing of a vanadium redox battery system for microgrid systems," *IEEE Transactions on Sustainable Energy*, vol. 6, pp. 729–737, 2015.

[17] Carpinelli G., Mottola F., and Proto D., "Probabilistic sizing of battery energy storage when time-of-use pricing is applied," *Electric Power Systems Research*, vol. 141, pp. 73–83, 2016.

[18] Li Y., Yang Z., Li G., Mu Y., Zhao D., Chen C., et al, "Optimal scheduling of isolated microgrid with an electric vehicle battery swapping station in multi-stakeholder scenarios: A bi-level programming approach via real-time pricing," *Applied Energy*, vol. 232, pp. 54–68, 2018.

[19] Li Y., Yang Z., Li G., Zhao D., and Tian W., "Optimal scheduling of an isolated microgrid with battery storage considering load and renewable generation uncertainties," *IEEE Transactions on Industrial Electronics*, vol. 66, pp. 1565–1575, 2019.

[20] Mbungu N. T., Bansal R. C., Naidoo R., Miranda V., and Bipath M., "An optimal energy management system for a commercial building with renewable energy generation under real-time electricity prices," *Sustainable Cities and Society*, vol. 41, pp. 392–404, 2018/08/01/ 2018.

[21] Nojavan S., Zare K., and Mohammadi-Ivatloo B., "Risk-based framework for supplying electricity from renewable generation-owning retailers to price-sensitive customers using information gap decision theory," International Journal of Electrical Power & Energy Systems, vol. 93, pp. 156–170, 2017.

[22] Nojavan S. and Zare K., "Interval optimization based performance of photovoltaic/wind/FC/electrolyzer/electric vehicles in energy price determination for customers by electricity retailer," *Solar Energy*, vol. 171, pp. 580–592, 2018.

[23] Sharma S., Bhattacharjee S., and Bhattacharya A., "Grey wolf optimisation for optimal sizing of battery energy storage device to minimise operation cost of microgrid," *IET Generation*, *Transmission & Distribution*, vol. 10, pp. 625–637, 2016.

[24] Sukumar S., Mokhlis H., Mekhilef S., Naidu K., and Karimi M., "Mix-mode energy management strategy and battery sizing for economic operation of grid-tied microgrid," *Energy*, vol. 118, pp. 1322–1333, 2017.

[25] Fossati J. P., Galarza A., Martín-Villate A., and Fontán L., "A method for optimal sizing energy storage systems for microgrids," *Renewable Energy*, vol. 77, pp. 539–549, 2015.

[26] Borhanazad H., Mekhilef S., Ganapathy V. G., Modiri-Delshad M., and Mirtaheri A., "Optimization of micro-grid system using MOPSO," *Renewable Energy*, vol. 71, pp. 295–306, 2014.

[27] Modiri-Delshad M., Kaboli S. H. A., Taslimi-Renani E., and Rahim N. A., "Backtracking search algorithm for solving economic dispatch problems with valve-point effects and multiple fuel options," *Energy*, vol. 116, pp. 637–649, 2016.

[28] Torreglosa J. P., Garcia P., Fernandez L. M., and Jurado F., "Predictive control for the energy management of a fuel-cell–battery–supercapacitor tramway," *IEEE Transactions on Industrial Informatics*, vol. 10, pp. 276–285, 2014.

[29] C. Ju and P. Wang, "Energy management system for microgrids including batteries with degradation costs," in Power System Technology (POWERCON), 2016 IEEE International Conference on, 2016, pp. 1–6.

[30] Han S., Han S., and Aki H., "A practical battery wear model for electric vehicle charging applications," *Applied Energy*, vol. 113, pp. 1100–1108, 2014.

[31] Zhou C., Qian K., Allan M., and Zhou W., "Modeling of the cost of EV battery wear due to V2G application in power systems," *IEEE Transactions on Energy Conversion*, vol. 26, pp. 1041–1050, 2011.

[32] Yang X.-S., Nature-inspired metaheuristic algorithms: Luniver press, 2010.

[33] Li Z., Zang C., Zeng P., Yu H., and Li S., "Agent-based distributed and economic automatic generation control for droop-controlled AC microgrids," *IET Generation*, *Transmission & Distribution*, vol. 10, pp. 3622–3630, 2016.

[34] Chen S., Gooi H. B., and Wang M., "Sizing of energy storage for microgrids," *IEEE Transactions on Smart Grid*, vol. 3, pp. 142–151, 2012.

[35] Ismail M., Moghavvemi M., and Mahlia T., "Techno-economic analysis of an optimized photovoltaic and diesel generator hybrid power system for remote houses in a tropical climate," *Energy Conversion and Management*, vol. 69, pp. 163–173, 2013.

