# Supplementary material for: Optimal sizing and energy scheduling of isolated microgrid considering the battery lifetime degradation
Source: PLoS One. 2019 Feb 14;14(2):e0211642. doi: 10.1371/journal.pone.0211642 (PMC6375580; doi:10.1371/journal.pone.0211642)
Supplement: S1 File — (PDF) [file pone.0211642.s001.pdf]

Hour	Load	Solar Power	Wind Power
1	36	0	37
2	30	0	37
3	35	0	30.4
4	40	0	28
5	55	0	26.3
6	75	0.1	35.4
7	96	5.3	37
8	100	15.9	27.1
9	132	29.2	22.2
10	155	41.4	33.7
11	163	53	30.4
12	145	57.5	28.8
13	120	52.7	35.4
14	92	42.1	30.4
15	76	49.9	32.9
16	68	46.4	28.8
17	66	29.7	27.1
18	74	20.4	20.6
19	103	11.9	18.9
20	104	2.1	24.7
21	100	0	27.1
22	82	0	32.1
23	76	0	37
24	64	0	37
